# Characterization and Chemoinformatic Prediction of Retention Indices of Metabolites in Coffee and Plantain Byproduct Flours Using Gas Chromatography–Time-of-Flight Mass Spectrometry

**DOI:** 10.1021/acs.jafc.5c11135

**Published:** 2025-11-17

**Authors:** Laura Sofía Torres-Valenzuela, Carolina Franco-Urbano, Diana Paola Navia-Porras, Nicole Sarmiento, Cristian Rojas

**Affiliations:** † Grupo de Investigación GIPAB, Escuela de Ingeniería de Alimentos, Universidad del Valle, Calle 13 No. 100-00, Cali 760032, Colombia; ‡ Grupo de Investigación Biotecnología, Facultad de Ingeniería, 27998 Universidad de San Buenaventura Cali, Carrera 122 # 6-65, Cali 76001, Colombia; § Grupo de Investigación en Quimiometría y QSAR, Facultad de Ciencia y Tecnología, 27892 Universidad del Azuay, Av. 24 de Mayo 7-77 Y Hernán Malo, Cuenca 010107, Ecuador

**Keywords:** chemoinformatics, coffee, GC-QTOF-MS, metabolites, plantain byproducts, QSRR, retention index

## Abstract

This study analyzed chemical metabolites in composite flours (CF) produced from coffee pulp, plantain rachis, and rejected plantain, as well as their changes induced by extrusion. Seventy-two metabolites were identified by gas chromatography–time-of-flight mass spectrometry (GC-QTOF-MS) analysis in an HP-5ms capillary column. Results revealed that extrusion induced a selective modification of the metabolites. Some antinutritional compounds (caffeine, deoxyglucose, and galactosamine) were reduced. These effects contributed to improving the nutritional and functional quality of the CF. The chromatographic information from a dataset of 265 molecules was used to develop a conformation-independent quantitative structure-retention relationship (QSRR) model with good predictive capability (*Q* ^2^ = 0.945), which was subsequently applied to estimate the retention index of the metabolites. This work provided an approach for metabolite profiling of agro-industrial CF and for predicting the chromatographic properties of new metabolites while advancing the valorization of coffee and plantain byproducts.

## Graphical abstract

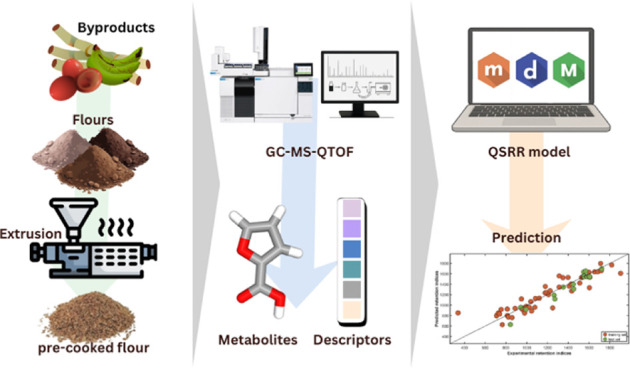

## 1 Introduction

The valorization of agro-industrial byproducts in the food industry has gained relevance as a strategy to increase sustainability and mitigate environmental impacts. In this context, coffee and plantain byproducts represent significant sources of biomass with a high utilization potential. Coffee pulp (CP), which accounts for approximately 45% of the fruit’s weight, is rich in carbohydrates, proteins, dietary fiber, and phytochemicals such as polyphenols and alkaloids. The levels of chlorogenic acid and epicatechin are particularly notable.^1^ Plantain cultivation generates large volumes of byproducts since only 20–30% of the biomass is processed.^2^ Two key byproducts from this production chain are plantain rachis (PR) and rejected plantain (RP) due to physical or pathological defects. The use of these three byproducts remains limited, as they are often underutilized or discarded in the soil. Such disposal alternatives contribute to environmental issues, such as soil and water contamination due to their phytotoxic effects.^3^ Nevertheless, these byproducts contain high levels of macronutrients and bioactive compounds such as anthocyanins and flavonoids, which have potential applications in the food and pharmaceutical industries.^4^ Integrating these byproducts into the formulation of precooked composite flour through technologies such as extrusion presents a viable alternative for developing value-added ingredients.

Extrusion is one of the most versatile unit operations in the agri-food industry. This process involves high-temperature short-time (HTST), cooking techniques and subjects the food matrix to mechanical shear, heat, and pressure. During extrusion, molecular alignment and chemical transformations increase the solubility, emulsification capacity, and lipid-carbohydrate complex formation, optimizing the uses of the final product.^5^ In addition to improving digestibility and nutritional value, extrusion inactivates non-nutritional compounds and reduces the microbial load, extending the shelf life of food products.^6^ Moreover, thermal processing can induce chemical reactions, such as the Maillard reaction and lipid oxidation, which modify the chemical profile and influence the sensory properties of the final product. Analysis of the metabolite composition of extruded composite flours provides insight into the chemical changes that are induced by thermal and mechanical processing and the impact of these changes on the sensory and functional qualities of the final product. There are recent studies of untargeted metabolomics to analyze changes in metabolites (amino acids, fatty acids, organic acids, and phenolic acids) induced by extrusion at different temperatures.^7,8^ Gas chromatography coupled with quadrupole time-of-flight mass spectrometry (GC-QTOF-MS) has emerged as a high-resolution analytical methodology that enables the precise identification and quantification of metabolites through accurate mass spectra and reference libraries.^9^ In GC-QTOF-MS analysis, the retention index (*RI*) is the primary analytical parameter that represents the relative elution time of a metabolite between two neighboring *n*-alkanes within the chromatogram. This parameter is independent of MS technology and is determined solely by chromatographic parameters. In temperature-programmed gas chromatography, the *RI* is useful for both analyzing and interpreting chromatographic information and also facilitates the reproducibility of GC analytical techniques under different conditions.^10^

The emerging impact of chemoinformatics in food chemistry, also known as foodinformatics,^11^ encompasses the profiling of bioactive compounds and predictive modeling, lipidome analysis, and species authentication in processed products, as well as applications in the development of sustainable pesticides and the improvement of crop yields.^12^ In this framework, quantitative structure-retention relationships (QSRRs) are well-established tools for analyzing chromatographic data from various food matrices. These QSRRs assume that a defined property (in this case, the retention index of flour metabolites) is correlated to molecular descriptors that represent different aspects of their chemical structure. Additionally, QSRR tools allow for better analysis and interpretation of the chromatographic phenomenon in such a way as to optimize the elucidation of new chemicals. Several QSRR studies have been published to model and predict the *RI* measured on the HP-5ms stationary phase in GC–MS, using compounds identified from food matrices and essential oils of flavoring additives. Table 1 summarizes the results of these models in a chronological way.

**Table 1:** Comparison of the Reported QSRR Models Retrieved from the Literature for Predicting the Retention Index for the HP-5ms Capillary Column

Model	QSRR model	Number of molecules	$R_{\mathrm{training}}^{2}$	RMSEC	$R_{\mathrm{test}}^{2}$	RMSEP
Riahi et al. (2009)^13^	SVM	100	0.987	24.7	0.962	51.4
Mohammadhosseini et al. (2011)^14^	MLR	43	0.984	29.9	0.954	49.3
Azar et al. (2011)^15^	MLR	25	0.983	27.8	0.970	34.5
Zhao et al. (2012)^16^	MSOP	178	0.999	36.0	–	32.6
Mohammadhosseini (2014)^17^	MLR	108	0.936	57.9	0.898	73.5
Nekoei and Mohammadhosseini (2014)^18^	MLR	27	0.886	87.6	0.890	103.0
Qin et al. (2013)^19^	MLR	169	0.936	89.0	0.923	–
Zanousi et al. (2017)^20^	MLR	67	0.978	–	0.986	25.4
Aćimović et al. (2020a)^21^	ANN	53	0.962	72.3	0.887	–
Aćimović et al. (2020b)^22^	ANN	26	0.968	394.6	0.704	478.5
Aćimović et al. (2021)^23^	BRT	160	–	–	0.956	66.2
			–	–	0.964	62.6
Lončar et al. (2025)^24^	ANN	47	0.980	1588.3	0.979	870.0
This work	MLR	265	0.945	73.8	0.945	67.4

The present study was designed to evaluate the use of three agro-industrial byproducts (CP, PR, and RP) to produce precooked composite flour via extrusion and to analyze the impact of this process on the nutritional properties of these products. This work focused on determining the composition of metabolites in the resulting flours via GC-QTOF-MS and the subsequent application of chemoinformatic approaches based on QSRR modeling to predict the retention indices (RIs) of these metabolites. The QSRR model was developed in alignment with the five principles set by the Organization for Economic Co-operation and Development. This model provides a robust framework for predicting the chromatographic retention patterns of metabolites in extruded coffee and plantain flours. To the best of the authors’ knowledge, no computer-based model has previously been developed to estimate the retention indices of compounds in mixed flours derived from coffee pulp and byproducts of plantains (plantain rachis and rejected plantains).

## 2 Materials and Methods

### 2.1 Analytical Determination of Metabolites in Extruded Flour Samples

#### 2.1.1 Raw Materials, Chemical Reagents, and Equipment

Coffee and plantain byproducts were obtained from Trujillo, Colombia (latitude 4° 12′ 41″ north, longitude 76° 19′ 13″ west, altitude 1317 masl). Coffee pulp (CP) was obtained from the mechanical pulping of mature “Castillo” coffee fruits. The plantain rachis (PR) and rejected plantains (RP) were collected manually after the plantains were harvested. Details of the equipment and materials are provided in the Supporting Information.

#### 2.1.2 Flour Processing and Extrusion Process

The PR and RP were shredded using a cutting machine. The PR, RP, and CP were dried at 45 °C for 19 h until they reached 10 ± 1% water content (wet basis, wb). This temperature was selected to simulate a solar drying process commonly used in Colombian coffee farms. The dried byproducts were milled and put through a 40-mesh sieve to produce flours (particle size < 0.425 mm). The flours were mixed as follows to produce a flour blend: 36.4% CP, 33.3% RP, and 30.3% PR. The proportion of flours was determined in a previous study using statistical optimization to maximize the protein content and the antioxidant properties in the formulation.^25^ The flour blend was divided into two fractions. The first fraction was considered the control, whereas the second fraction was subjected to an extrusion process. Before extrusion, the flour blend was adjusted to a moisture content of 18% (wb). The extrusion was carried out in a twin-screw extruder with three heating zones set to 100, 160, and 120 °C. The values for the moisture content of the mixture and the temperature profile were chosen to confirm those reported in previous studies on cereal flours.^26,27^ The outlet nozzle had a 5 mm internal diameter, and the input and output power supply frequencies were 18 and 36 Hz, respectively. After processing, the extrudates (<11.5% moisture, wb) were cooled for 4 h, milled, and sieved through a 40-mesh sieve. The control and extruded flours were stored in sealed polypropylene bags at room temperature for further analysis.

#### 2.1.3 GC-QTOF-MS Analysis

For the extraction of metabolites, 30 mg of sample was mixed with 500 μL of MeOH (−20 °C). The mixture was vortexed (10 min), sonicated (10 min), and then vortexed again (5 min). The samples were filtered through 0.22 μm filters. From the previously prepared extracts, 70 μL was dried in a SpeedVac for 1 h at 35 °C. The dried extracts were subjected to methoximation and silylation to derive polar groups and thus increase compound volatility and detectability. For this purpose, 10 μL of *O*-methoxylamine in pyridine (15 mg/mL) was added, and the mixture was vortexed at 3200 rpm for 5 min and incubated in the dark at room temperature for 16 h. The silylation process was carried out by adding 10 μL of *N*,*O*-bis­(trimethylsilyl)­fluoroacetamide (BSTFA) with 1% trimethylchlorosilane (TMCS), vortexing for 5 min, and incubating at 70 °C for 1 h. The samples were allowed to cool to room temperature for 30 min before 50 μL of methyl stearate in heptane was added as an internal standard (5 mg/L), and then the samples were vortexed for 5 min at 3200 rpm. Metabolites are reported in their underivatized form.

For data acquisition, a gas chromatograph system coupled to a time-of-flight mass selective detector equipped with a split/splitless injection port (250 °C, divided ratio 50) was employed. The electron ionization (EI) source was operated at 70 eV. A J&W HP-5ms column (30 m × 0.25 mm, 0.25 μm), with a (5% phenyl)-methylpolysiloxane, was used, with helium as the carrier gas at a constant flow rate of 0.7 mL/min. The oven temperature was programmed to start at 60 °C (1 min), increasing to 325 °C at a rate of 10 °C/min (10 min). The transfer line, ion source filament, and quadrupole temperatures were maintained at 280, 230, and 150 °C, respectively.

MS detection was performed in the 50–600 *m*/*z* range at 5 spectra/s. Deconvolution, alignment, and integration were carried out using Agilent Unknowns Analysis B.10.0, MassProfiler Professional v15, and Agilent MassHunter Quantitative Analysis B.10.00 software, respectively. Metabolite identification was performed using the “Fiehn GC-MS Metabolomics RTL Library” (2011 version), which considers retention time matching, mass spectra, and retention indices (*RIs*) of fatty acid methyl esters (FAMEs) or alkanes (C7–C40). Mass spectral similarity was evaluated using a ±10 ppb tolerance. These metabolites were assigned a level 2 identification according to the Metabolomics Standards Initiative.^28^ Information about matching scores is presented in Table S1 in the Supporting Information.

Quality controls (QCs) were prepared by mixing equal volumes of each sample and analyzing them following the previously described procedures. Multiple QC runs were performed to ensure system stability and reproducibility before random analysis throughout the sequence.

#### 2.1.4 Statistical Characterization of Metabolites

Based on the amount of the metabolites in coffee and plantain byproduct flours before and after extrusion, a comparative multiple-stage statistical analysis was conducted. The analysis was conducted for exploratory purposes to visualize group separation and identify discriminant metabolites. Initially, the data were normalized using the systematic error removal method with a Random Forest-based normalization algorithm. Then, normalization was performed based on the total ion area of each sample and the area of the internal standard. After normalization, presence and variability filters were applied, retaining only those features detected in 100% of the samples and exhibiting a coefficient of variation (CV) below 20% in quality control (QC) samples. Furthermore, the orthogonal partial least-squares discriminant analysis (OPLS-DA) was employed to obtain the variable importance in projection (VIP) scores and the corresponding confidence intervals using the Jackknife resampling method. The quality parameters were *R* ^2^ = 0.88 and *Q* ^2^ = 0.84. Finally, the *t*-Student test was conducted for group comparison. Multiple testing correction was applied using the Benjamini–Hochberg false discovery rate (FDR) approach. Metabolites were considered statistically significant if at least one of the following conditions was satisfied: (1) *p*-value is lower than 0.05; or (2) VIP score is greater than 1. All of the statistical analyses were performed in the MetaboAnalyst 5.0 software.^29^

### 2.2 Chemoinformatic Prediction of Retention Indices of Metabolites in Extruded Flour Samples

#### 2.2.1 Database Generation and Molecular Feature Representation

To predict the retention indices of metabolites identified in flour samples in the HP-5ms capillary column, an external dataset comprising 310 flavor compounds reported by Yan and coworkers^30^ was used to calibrate the model. For each compound, the PubChem CID (Compound Identifier) and simplified molecular input line entry system (SMILES) were retrieved from the PubChem open-access library.^31^ The SMILES notation was then used to curate molecular structures and to filter the dataset. The curation process checked for chemical errors and standardized chemotypes to ensure accurate chemical representation of the compounds. The filtering process used a software-implemented option that applies chemical formula matching to identify and merge duplicated molecules before calculating conformation-independent molecular descriptors (MDs). A molecular descriptor is a numerical value derived through well-defined rules (invariants) that represent the chemical structure of a metabolite. Molecular descriptors are used to develop reliable quantitative structure-retention relationship (QSRR) models.^32^ In this work, 4182 conformation-independent molecular descriptors and 166 Molecular ACCess System (MACCS) structural keys were calculated.

#### 2.2.2 Development and Validation of the QSRR Model

For the development of the QSRR model, the dataset was divided into training and test sets. The splitting process is fundamental for the proper assignment of molecules to both sets. Partitioning the dataset allows for the examination of model performance during both calibration and prediction, along with cross-validation. In this study, the dataset was randomly divided into training and test sets at a 70:30 ratio. To calibrate the QSRR model, the training set molecules were used to search for the most suitable MDs for inclusion in the optimal QSRR model, ensuring good performance in cross-validation and, most importantly, strong predictive capability. For the supervised selection of the most informative molecular features, the genetic algorithm-variable subset selection (GA-VSS) method was used^33^ coupled with multiple linear regression (MLR) based on the ordinary least-squares (OLS) approach. GA-VSS operates by creating a pool of models (also called chromosomes), where the absence (0) or presence (1) of molecular descriptors is defined in a binary vector. Then, GA-VSS generates new QSRR models by combining the initial chromosomes through a crossover mechanism and/or randomly including or excluding molecular features using a probabilistic rule (mutation mechanism). The GA-VSS process should be repeated as many times as possible (for instance, 100 runs). During supervised feature selection, the 5-fold Venetian blinds method (leave-many-out with 20%) was used to avoid overfitting the model by optimizing the root-mean-squared error (RMSECV) during cross-validation.

The optimal model was further analyzed to ensure its stability by predicting the *RI* values of molecules that were excluded from the training set using diverse cross-validation criteria. These included leave-one-out (LOO), 5-fold Venetian blinds, continuous blocks, Monte Carlo, and bootstrap methods. A well-calibrated QSRR model should exhibit a balance between the coefficient of determination (*R* ^2^) and the RMSE across both calibration and cross-validation. Additionally, the Y-randomization approach^34^ was used to verify the absence of chance correlation by randomly permutating the experimental *RI* values in the training set multiple times (e.g., 1000 runs). In this case, the *R*_cv_^2^ and RMSECV statistical parameters should be significantly different from those of the calibrated model. Finally, the test set compounds, which were never used during model optimization and calibration, were used to assess the predictive ability of the *in silico* model. The predictive capability of the model was evaluated using the *Q* ^2^ metric (specifically *Q*_F3_^2^) and the root mean squared error of prediction (RMSEP).^35^

#### 2.2.3 Mechanism of Action of the QSRR Model

The reliability of a QSRR model is also related to the mechanism of action of the molecular descriptors used for model calibration; that is, an explanation of how those MDs are related to retention index prediction. This issue is important, particularly for reproducibility and regulatory purposes.^36,37^ Importantly, the calculation of some molecular descriptors involves the use of complex algorithms, and it is therefore challenging to provide a full explanation of the descriptor itself and the way in which it is related to the *RI* prediction. On the other hand, other descriptors are easy to explain in such a way as to construct a causality effect between the *RI* property and the features used to calibrate the *in silico* model. In the framework of MLR models, the best way to analyze the contributions of MDs is by standardizing the regression coefficients. Thus, it is possible to quantify a descriptor’s contribution and how it is related to the retention index when the other MDs remain constant. Additionally, the synergistic or antagonistic effect of each descriptor in predicting the *RI* property is related to the positive or negative sign, respectively. The mechanism of action of MDs contributes to a chemical understanding of the interactions between flavor compounds during chromatographic separation and complements experimental chemists’ work during the optimization of chromatographic analysis.

#### 2.2.4 Applicability Domain Assessment of the QSRR Model

For regulatory purposes, the reliability of the QSRR model for predicting the *RI* of new compounds is related to the definition of the applicability domain (AD). The AD is a theoretical chemical space that is defined by the molecular features used to calibrate the model, enabling reliable predictions for new compounds based on their similarity to the training metabolites used to optimize the model. In MLR *in silico* models, the leverage approach is the most common method for defining this theoretical chemical space.^38^ This approach uses the Hat matrix (**Ĥ**), whose diagonal elements (*h*_*ii*_) correspond to the leverage values of the compounds. Additionally, a threshold value (also known as the warning leverage) is calculated as three times the number of parameters of the model divided by the total number of molecules used in the training set to calibrate the model. The leverage value for each test set compound is also computed. A prediction is considered reliable (model interpolation) if the *h*_*ii*_ value of a compound in the test set is lower than the threshold leverage (h*); otherwise, the *RI* prediction is deemed unreliable, as the test compound falls far from the centroid of the AD, indicating model extrapolation. The results of the AD chemical space analysis are presented in a Williams plot, which presents the leverage values and standardized residuals of the molecules.

### 2.3 Software

The chemical structures of the molecules were visualized using MarvinSketch software.^39^ The structures were verified and curated with alvaMolecule,^40,41^ which was also employed to merge conformation-independent molecules. Molecular descriptors were calculated and reduced using the V-WSP algorithm in alvaDesc.^40,41^ Dataset splitting, model calibration, validation, applicability domain assessment, and application of the QSRR model to metabolites of flours were performed with alvaModel.^40,41^

## 3 Results and Discussion

### 3.1 Analytical Determination of Metabolites in Extruded Flour Samples

GC analysis indicated that the extrusion process differentially affected the compounds that are present in the composite flour. The cluster in the principal component analysis (PCA) for the QC samples (gray dots) indicates high analytical reproducibility (Figure S1a). Additionally, a clear separation between extruded and control samples was observed, which suggests distinct chemical composition. This trend was supported by the OPLS-DA models (Figure S1b), which also revealed a separation between groups.

Table 2 presents the identified metabolites, and Table 3 shows the relative abundance of each compound before and after extrusion and their change. While the levels of some metabolites significantly increased, indicating possible degradation reactions or the formation of new products, the levels of others remained unchanged. The analysis of changes in the composition of the extruded flour was conducted based on the change in the relative abundance (fold-change) of the compound (estimated from its chromatographic peak area). The analysis of extruded and nonextruded composite flour revealed significant changes in the relative abundance of various metabolites, indicating potential effects of extrusion on the flour matrix. The stability of alcohols and polyols suggested that the extrusion conditions did not induce thermal degradation or chemical transformation. Similarly, most amino acids remained intact, indicating minimal deamination or Maillard reactions. While the relative abundance of some carbohydrates and sugars was slightly reduced, there was no significant evidence of caramelization or extensive degradation.

**Table 2:** Metabolite Composition of Coffee and Plantain Byproduct Flours Identified by GC-QTOF-MS Using an HP-5ms Capillary Column

				Retention index (*RI*)		
No.	Metabolite	CAS registry number	Retention time (*t*_R_) min	Experimental	Literature^a^	Chemical class
1	erythritol	149-32-6	13.237	1193	–	Alcohols and Polyols
2	2,3-butanediol	513-85-9	6.570	709	782	Alcohols and Polyols
3	glycerol	56-81-5	10.173	960	–	Alcohols and Polyols
4	arabitol	488-82-4	15.630	1409	–	Alcohols and Polyols
5	diglycerol	627-82-7	15.947	1428	–	Alcohols and Polyols
6	ethanolamine	141-43-5	10.059	952	–	Amino Acids and Derivatives
7	glycine	56-40-6	10.637	993	–	Amino Acids and Derivatives
8	cycloleucine	52-52-8	11.344	1046	–	Amino Acids and Derivatives
9	glutamic acid	56-86-0	13.446	1210	–	Amino Acids and Derivatives
10	glucosamine-phosphate	2152-75-2	16.619	1492	–	Amino Acids and Derivatives
11	galactosamine	90-76-6	17.232	1551	–	Amino Acids and Derivatives
12	tyramine	51-67-2	17.789	1604	1660.9^b^	Amino Acids and Derivatives
13	glucosaminic acid	3646-68-2	18.567	1685	–	Amino Acids and Derivatives
14	valine	72-18-4	7.366	764	–	Amino Acids and Derivatives
15	valeramide	626-97-1	8.207	823	–	Amino Acids and Derivatives
16	isoleucine	443-79-8	8.716	858	–	Amino Acids and Derivatives
17	serine	56-45-1	9.904	941	–	Amino Acids and Derivatives
18	pipecolic acid	535-75-1	9.993	948	–	Amino Acids and Derivatives
19	threonine	72-19-5	10.440	979	–	Amino Acids and Derivatives
20	malonamide	108-13-4	11.751	1078	–	Amino Acids and Derivatives
21	acetyl-glutamic acid	1188-37-0	13.049	1179	–	Amino Acids and Derivatives
22	ornithine	70-26-8	14.158	1271	–	Amino Acids and Derivatives
23	2-deoxy-ribose	533-67-5	13.790	1226	–	Carbohydrates and Sugars
24	deoxyglucose	154-17-6	16.150	1448	–	Carbohydrates and Sugars
25	1,5-anhydroglucitol	154-58-5	16.892	1187	–	Carbohydrates and Sugars
26	methyl-galactopyranoside	3396-99-4	16.992	1528	–	Carbohydrates and Sugars
27	galactonic acid	576-36-3	17.216	1549	–	Carbohydrates and Sugars
28	sorbose	87-79-6	17.229	1546	–	Carbohydrates and Sugars
29	galactose	59-23-4	17.235	1551	–	Carbohydrates and Sugars
30	mannose	3458-28-4	17.243	1551	–	Carbohydrates and Sugars
31	sedoheptulose anhydride monohydrate	469-90-9	17.740	1599	–	Carbohydrates and Sugars
32	glucose	2280-44-6	17.830	1608	–	Carbohydrates and Sugars
33	glucoheptonic acid	87-74-1	18.834	1712	–	Carbohydrates and Sugars
34	mucic acid	526-99-8	18.885	1717	–	Carbohydrates and Sugars
35	1,5-anhydrosorbitol	154-58-5	13.154	1511	–	Carbohydrates and Sugars
36	xylose	25990-60-7	14.795	1313	–	Carbohydrates and Sugars
37	ribose	50-69-1	15.096	1352	–	Carbohydrates and Sugars
38	fructose	57-48-7	17.558	1582	–	Carbohydrates and Sugars
39	palmitic acid	57-10-3	18.931	1722	1917, 1929, 1952, 1953, 1957, 1962, 1969, 1973, 1975	Lipids
40	nonanoic acid	112-05-0	11.248	1039	1270, 1271, 1297	Lipids
41	heptadecanoic acid	506-12-7	19.843	1819	2077	Lipids
42	2-furoic acid	88-14-2	8.074	813	–	Organic Acids
43	malonic acid	141-82-2	9.130	887	–	Organic Acids
44	fumaric acid	110-17-8	11.083	1025	–	Organic Acids
45	hydroxyhexanoic acid	1191-25-9	11.823	1083	–	Organic Acids
46	malic acid	6915-15-7	13.028	1177	–	Organic Acids
47	hydroxyglutaric acid	2889-31-8	14.065	1263	–	Organic Acids
48	aconitic acid	585-84-2	16.017	1434	–	Organic Acids
49	dihydroxybenzoic acid	99-50-3	16.815	1511	–	Organic Acids
50	quinic acid	77-95-2	17.415	1568	–	Organic Acids
51	gluconic acid lactone	90-80-2	17.556	1581	–	Organic Acids
52	glucuronolactone	32449-92-6	17.820	1608	–	Organic Acids
53	gallic acid	149-91-7	18.260	1653	–	Organic Acids
54	shikimic acid	138-59-0	20.609	1905	2036.6^c^	Organic Acids
55	pyruvic acid	127-17-3	6.812	725	–	Organic Acids
56	lactic acid	79-33-4	6.992	738	–	Organic Acids
57	glycolic acid	79-14-1	7.203	753	–	Organic Acids
58	oxalic acid	144-62-7	8.059	812	–	Organic Acids
59	hydroxybutyric acid	300-85-6	8.487	842	–	Organic Acids
60	nicotinic acid	59-67-6	10.384	975	–	Organic Acids
61	succinic acid	110-15-6	10.655	994	–	Organic Acids
62	glyceric acid	473-81-4	10.972	1017	–	Organic Acids
63	dihydroxymalonic acid	560-27-0	12.313	1121	–	Organic Acids
64	3-hydroxypropanoic acid	503-66-2	13.911	1250	–	Organic Acids
65	hydroxybenzoic acid	99-96-7	14.637	1313	1577	Organic Acids
66	vanillic acid	121-34-6	16.195	1452	1608	Organic Acids
67	gulonic acid lactone	1128-23-0	17.761	1601	–	Organic Acids
68	caffeine	58-08-2	17.130	1540	–	Alkaloids
69	1,2-dihydro-1,2-naphthalenediol	31966-70-8	14.658	1313	–	Phenolic Compounds
70	4-O-methylphloracetophenone	7507-89-3	15.627	1398	–	Phenolic Compounds
71	1,3-dihydroxyacetone	96-26-4	9.685	926	–	Hydroxy Ketones
72	phosphoric acid	7664-38-2	10.178	960	–	Inorganic Acid

a Retention index retrieved from the National Institute of Standards and Technology (NIST), U.S. Department of Commerce: https://www.nist.gov/.

b not available.

c Van Den Dool and Kratz retention index.

**Table 3:** Details of Metabolite Changes of Coffee and Plantain Byproduct Flours before and after Extrusion

		Relative abundance					
No.	Metabolite	Before extrusion	After extrusion	Fold change (log_2_ scale)	Description	*p*-value	Variable importance in correction
1	erythritol	0.0396	0.0214	–0.89	Decrease	1.78 × 10^–02^ ^a^	1.09
2	2,3-butanediol	0.2532	0.1340	–0.92	No change	>0.05	<1.00
3	glycerol	0.9457	0.5934	–0.67	No change	>0.05	<1.00
4	arabitol	0.0130	0.0071	–0.86	No change	>0.05	<1.00
5	diglycerol	0.0169	0.0054	–1.64	No change	>0.05	<1.00
6	ethanolamine	0.0007	0.0391	5.90	Increase	2.97 × 10^–04^ ^a^	1.37
7	glycine	0.0023	0.0165	2.82	Increase	5.83 × 10^–04^ ^a^	1.33
8	cycloleucine	0.0193	0.0087	–1.15	Decrease	1.91 × 10^–02^ ^a^	1.09
9	glutamic acid	3.1486	0.9798	–1.69	Decrease	6.02 × 10^–05^ ^a^	1.41
10	glucosamine-phosphate	0.0168	0.0065	–1.40	Decrease	2.26 × 10^–03^ ^a^	1.29
11	galactosamine	0.0331	0.0023	–3.84	Decrease	3.06 × 10^–04^ ^a^	1.34
12	tyramine	0.0023	0.0678	4.89	Increase	2.70 × 10^–03^ ^a^	1.27
13	glucosaminic acid	11.9755	1.3405	–3.18	Decrease	1.06 × 10^–02^ ^a^	1.15
14	valine	0.0434	0.0279	–0.64	No change	>0.05	<1.00
15	valeramide	0.2337	0.1664	–0.49	No change	>0.05	<1.00
16	isoleucine	0.0129	0.0062	–1.06	No change	>0.05	<1.00
17	serine	0.0172	0.0143	–0.27	No change	>0.05	<1.00
18	pipecolic acid	0.0060	0.0066	0.14	No change	>0.05	<1.00
19	threonine	0.0051	0.0030	–0.79	No change	>0.05	<1.00
20	malonamide	0.0015	0.0022	0.56	No change	>0.05	<1.00
21	acetyl-glutamic acid	0.0891	0.0284	–1.64	No change	>0.05	<1.00
22	ornithine	0.0357	0.0249	–0.51	No change	>0.05	<1.00
23	2-deoxy-ribose	0.0323	0.0134	–1.25	Decrease	7.73 × 10^–03^ ^a^	1.17
24	deoxyglucose	0.0148	0.0046	–1.69	Decrease	1.04 × 10^–03^ ^a^	1.33
25	1,5-anhydroglucitol	0.0073	0.0078	0.08	Increase	2.71 × 10^–02^	1.04
26	methyl-galactopyranoside	4.2884	0.0147	0	Decrease	4.92 × 10^–03^ ^a^	1.21
27	galactonic acid	0.1845	0.0116	–4.06	Decrease	1.37 × 10^–03^ ^a^	1.30
28	sorbose	0.2021	0.0091	–4.64	Decrease	7.67 × 10^–03^ ^a^	1.18
29	galactose	0.2058	0.0114	–4.06	Decrease	1.24 × 10^–02^ ^a^	1.12
30	mannose	0.0380	0.0033	–3.47	Decrease	1.24 × 10^–05^ ^a^	1.42
31	sedoheptulose anhydride monohydrate	11.1883	0.2812	–5.06	Decrease	6.72 × 10^–04^ ^a^	1.33
32	glucose	65.2808	27.9535	–1.22	Decrease	1.52 × 10^–02^ ^a^	1.10
33	glucoheptonic acid	3.8824	1.5047	–1.36	Decrease	6.37 × 10^–05^ ^a^	1.41
34	mucic acid	0.7020	0.1846	–1.94	Decrease	5.81 × 10^–03^ ^a^	1.20
35	1,5-anhydrosorbitol	2.0258	0.0109	–6.64	No change	>0.05	<1.00
36	xylose	0.0192	0.0113	–0.76	No change	>0.05	<1.00
37	ribose	0.0080	0.0028	–1.51	No change	>0.05	<1.00
38	fructose	53.5614	27.4699	–0.97	No change	>0.05	<1.00
39	palmitic acid	0.7225	0.4756	–0.60	Decrease	2.81 × 10^–02^	1.04
40	nonanoic acid	0.0044	0.0026	–0.76	No change	>0.05	<1.00
41	heptadecanoic acid	0.0022	0.0016	–0.47	No change	>0.05	<1.00
42	2-furoic acid	0.0013	0.0034	1.42	Increase	2.23 × 10^–03^ ^a^	1.28
43	malonic acid	0.0808	0.0365	–1.15	Decrease	3.86 × 10^–02^	<1.00
44	fumaric acid	0.0870	0.0292	–1.56	Decrease	2.47 × 10^–02^	1.05
45	hydroxyhexanoic acid	0.1176	0.0369	–1.69	Decrease	1.31 × 10^–03^ ^a^	1.30
46	malic acid	1.3460	0.4315	–1.64	Decrease	2.72 × 10^–04^ ^a^	1.37
47	hydroxyglutaric acid	0.0064	0.0018	–1.84	Decrease	3.00 × 10^–03^ ^a^	1.24
48	aconitic acid	0.0026	0.0012	–1.18	Decrease	3.17 × 10^–04^ ^a^	1.35
49	dihydroxybenzoic acid	0.0857	0.0272	–1.64	Decrease	3.95 × 10^–05^ ^a^	1.42
50	quinic acid	12.0297	2.7977	–2.12	Decrease	7.00 × 10^–05^ ^a^	1.40
51	gluconic acid lactone	0.1219	0.0498	–1.29	Decrease	3.37 × 10^–02^	1.01
52	glucuronolactone	83.1777	38.6568	–1.12	Decrease	3.90 × 10^–02^	<1.00
53	gallic acid	0.0172	0.0046	–1.94	Decrease	3.43 × 10^–07^ ^a^	1.47
54	shikimic acid	1.0267	0.1383	–2.94	Decrease	3.38 × 10^–05^ ^a^	1.41
55	pyruvic acid	0.0139	0.0118	–0.23	No change	>0.05	<1.00
56	lactic acid	1.0819	0.7853	–0.45	No change	>0.05	<1.00
57	glycolic acid	0.0267	0.0226	–0.23	No change	>0.05	<1.00
58	oxalic acid	2.2650	1.4398	–0.64	No change	>0.05	<1.00
59	hydroxybutyric acid	0.0017	0.0013	–0.34	No change	>0.05	<1.00
60	nicotinic acid	0.0049	0.0020	–1.32	No change	>0.05	<1.00
61	succinic acid	0.7125	0.3913	–0.86	No change	>0.05	<1.00
62	glyceric acid	0.0630	0.0325	–0.94	No change	>0.05	<1.00
63	dihydroxymalonic acid	0.0024	0.0025	0.08	No change	>0.05	<1.00
64	3-hydroxypropanoic acid	0.0099	0.0055	–0.84	No change	>0.05	<1.00
65	hydroxybenzoic acid	0.0027	0.0026	0.00	No change	>0.05	<1.00
66	vanillic acid	0.0019	0.0021	0.19	No change	>0.05	<1.00
67	gulonic acid lactone	6.9292	1.4171	–2.32	No change	>0.05	<1.00
68	caffeine	1.8938	1.0039	–0.92	Decrease	2.81 × 10^–03^ ^a^	1.26
69	1,2-dihydro-1,2-naphthalenediol	0.0049	0.0048	–0.04	No change	>0.05	<1.00
70	4-O-methylphloracetophenone	0.0018	0.0020	0.14	No change	>0.05	<1.00
71	1,3-dihydroxyacetone	0.0196	0.0116	–0.76	No change	>0.05	<1.00
72	phosphoric acid	2.6289	0.9594	–1.47	Decrease	2.88 × 10^–03^ ^a^	1.24

a Calculated by the Benjamini–Hochberg false discovery rate post hoc correction.

Additionally, the relative abundance of phenolic compounds remained stable, preserving their antioxidant properties. These findings indicate that the extrusion parameters were effectively controlled, preventing excessive heat-induced degradation and ensuring the retention of the nutritional and functional properties of the final product.

In addition to their nutritional and bioactive potential, the extrusion induced changes in some antinutritional compounds. The concentration of oxalic acid remained unchanged, suggesting that this compound is relatively stable under the applied processing conditions. In contrast, deoxyglucose, galactosamine, and caffeine decreased after extrusion (0.31-, 0.07-, and 0.53-fold changes respectively). These reductions may contribute to improving the nutritional quality of the composite flour.

Oxalic acid is known to form insoluble complexes with minerals such as calcium and iron, reducing their bioavailability;^42^ deoxyglucose is a nonmetabolizable glucose analogue^43^ that may interfere with energy metabolism under specific conditions; galactosamine has been reported to induce hepatotoxicity by blocking RNA and protein synthesis and by triggering oxidative stress and inflammatory responses;^44^ and caffeine may exert antinutritional effects by inhibiting the intestinal absorption of essential minerals such as iron and zinc.^45^ The results are discussed by metabolite type as follows:

#### 3.1.1 Amino Acids and Derivatives

Ethanolamine is a key component of phospholipid metabolism, and increases in its relative abundance could indicate increased membrane lipid turnover.^46^ Similarly, tyramine is known to arise from amino acid decarboxylation during thermal processing.^47^ A significant increase in the relative abundance of glycine (7.1) may be associated with protein or peptide hydrolysis, which has been reported in extruded wheat gluten.^48^ The stability of valine, isoleucine, serine, and threonine levels under the extrusion conditions that were used in this study suggests that these amino acids may be less sensitive to thermal degradation, corroborating findings in maize and rice brands.^49^ These changes can influence the nutritional profile of extruded products, potentially improving protein digestibility and bioavailability. Cui et al.,^48^ and Song and Tang^7^ reported the impact of extrusion on the content of some amino acids; compounds such as l-serine increased, while l-aspartic acid decreased. These results suggest differential thermal stability and transformation of amino acids, as well as structural modifications that enhance enzymatic hydrolysis and alter amino acid profiles.

#### 3.1.2 Carbohydrates and Sugars

Most simple sugars (fructose, ribose, and xylose) did not undergo significant changes after extrusion. However, the relative abundance of some compounds, such as 2-deoxy-ribose (0.4), glucose (0.4), and deoxyglucose (0.4), showed moderate reductions, suggesting partial degradation or conversion into other derivatives, possibly due to caramelization or Maillard reactions. These findings suggest that certain sugars may be susceptible to the high temperatures and pressures of the extrusion process, which can be associated with differences in the thermal properties and molecular weights of the sugars.^50^

#### 3.1.3 Organic Acids

Increases in the levels of several organic acids, particularly 2-furoic acid (2.7), were detected, which may indicate the formation of Maillard reaction products or carbohydrate degradation. The levels of other compounds, such as malonic acid (0.5) and fumaric acid (0.3), decreased, which is possibly related to the thermal and mechanical effects of extrusion.^51,52^ Although specific information on the impact of extrusion on organic acids is limited, related studies suggest that extrusion can induce chemical changes in quinoa flour, resulting in significant reductions in the contents of furoic acid, shikimic acid, succinic acid, hydroxybenzoic acid, and vanillic acid; however, higher values were observed for malic acid and quinic acid,^7^ suggesting that extrusion can alter specific compounds in flours. These changes influence flavor by modifying acidity (sour taste), balancing bitter or umami notes, reducing off-flavors, and finally improving palatability in extruded products.^7,53^

#### 3.1.4 Fatty Acids

The relative abundance of palmitic acid (C16:0) decreased slightly (0.8), which could be associated with the breakdown of triglycerides and the release of free fatty acids. Although heptadecanoic (C17:0) and nonanoic (C9:0) acids are also saturated, their relative abundance remained relatively stable. This behavior may be related to the positional distribution within triacylglycerols, as C16:0 is preferentially located at the sn-1 and sn-3 positions, which are more prone to thermal hydrolysis.^54^ In contrast, the lower abundance and possible sn-2 or less reactive positioning of C17:0 and C9:0 may contribute to their thermal stability. Previously, it was reported that a similar selective decrease of C16:0 in heat-treated oils occurred.^55^

Interestingly, odd-chain fatty acids, such as heptadecanoic (C17:0) and nonanoic (C9:0) acids, were detected in this matrix, despite being uncommon in plant materials. Their presence has been reported at trace levels in certain vegetable oils and tissues and has even been associated with specific stages of plant development^56,57^ or microbial activity during postharvest handling.^58^ Spontaneous fermentation prior to the drying of coffee pulp could lead to the presence of these compounds.

#### 3.1.5 Other Compounds

A decrease in caffeine concentration (0.5) was observed, which could be related to the release of this compound from the food matrix or its concentration due to the loss of other compounds. The relative abundances of phenolic compounds and hydroxy ketones were not affected. The reduction in caffeine concentrations during extrusion may be beneficial, as it decreases its inhibitory effect on the absorption of minerals such as iron and zinc.^45^ In contrast, the stability of phenolic compounds may not be entirely favorable, as this group includes tannins, which are known to bind to proteins and inhibit digestive enzymes such as trypsin and amylase, thereby reducing nutrient availability. Duguma et al.^6^ reported that extrusion led to significant reductions in antinutritional factors such as phytic acid and condensed tannins, which were attributed to thermal degradation and matrix modification. On the other hand, extrusion temperatures promote the release of bound phenolic compounds, maintaining the antioxidant potential in the extruded product.^59^

In summary, the relationship between metabolite changes and nutritional quality is positive, as extrusion did not significantly affect key nutritional compounds, including amino acids, fatty acids, carbohydrates, and phenolic compounds. Similarly, in studies of underutilized cereals,^60^ peanut extrudates,^61^ mixtures of roasted coffee powder in whole grain sorghum flours,^62^ and wheat-based extrudates with germinated cowpea flour,^63^ it was determined that the changes generated by extrusion improved the nutritional quality of the products obtained.

The findings of this work enhance the potential for future technological applications of this extruded flour in both human and animal feed. The resulting products should undergo sensory evaluations to assess the impact of partially or fully using this blend on the flavor, aroma, and palatability of the final consumable products. Moreover, it is necessary to evaluate the use of the extruded flour in the formulation of new food products and conduct comprehensive nutritional and sensory evaluations to determine their technological performance, acceptability, and potential benefits for targeted populations. In this context, the QSRR model developed in this work offers a powerful tool to predict the presence of metabolites in these food matrices, which may facilitate future research efforts and reduce the cost associated with chemical analysis.

### 3.2 Chemoinformatic Prediction of Retention Indices of Metabolites in Extruded Flour Samples

#### 3.2.1 Development and Validation of the QSRR Model

For the development of the *in silico* model for predicting the retention index (*RI*) of metabolites listed in Table 2, we considered the 310 flavor compounds from Yan and coworkers’ database^30^ to calibrate a conformation-independent quantitative structure-retention relationship (QSRR). The SMILES criterion was applied to identify duplicate compounds across the two datasets. As a result, 265 unique compounds from Yan and coworkers’ database were used for model calibration (refer to Table S2), while 67 metabolites were employed for model prediction (refer to Table S3). For merged molecules, the average *RI* value was used for both model development and model application. Molecules were chemically represented by 4182 conformation-independent molecular descriptors and 166 Molecular ACCess System (MACCS) structural keys. Subsequently, 2071 descriptors with constant values and 91 molecular features with missing values were eliminated prior to model optimization through supervised variable selection. As a result, 2020 molecular descriptors, which were grouped into 21 descriptor blocks, were retained for chemoinformatic modeling. For model development, the dataset of 265 compounds was randomly divided into training and test sets, consisting of 186 and 79 molecules, respectively. Compounds with low and high retention indices were retained in the training set. The compounds assigned to the training and test sets are listed in Table S2 of the Supporting Information. Training molecules were used for model optimization and cross-validation, and the test set was used to evaluate the predictive performance of the optimized model. For model optimization, genetic algorithm-variable subset selection (GA-VSS) was performed in two stages to manage the 2020 molecular features. First, GA-VSS was applied separately to each of the 21 descriptor blocks. Second, the top-performing descriptors were merged into a dataset of 479 descriptors, and GA-VSS was applied again to define the optimal model. During optimization, the root mean squared error in 5-fold cross-validation (RMSECV) was used as the performance score. Using this approach, a three-descriptor chemoinformatic model was identified, as presented in eq 1

1 RI=428.2+126.7Eta_betaS+13.8MDEC‐22+627.1MATS1p

The developed QSRR model included three descriptors that exhibited a synergistic effect on *RI* prediction; that is, the greater their values were, the higher the predicted *RI*. These features were: the eta sigma VEM count (Eta_betaS), the molecular distance edge between all secondary carbons (MDEC-22) and the Moran autocorrelation of lag 1 weighted by polarizability (MATS1p). The values for the three molecular descriptors and the predicted retention indices are presented in Table S2 of the Supporting Information.

The quantitative structure-property relationship described in eq 1 demonstrated good performance in calibration (*R* ^2^ = 0.945 and RMSEC = 73.8), which was confirmed with diverse cross-validation protocols. The internal robustness of the chemoinformatic model was further confirmed using six well-known cross-validation approaches: leave-one-out (*R*_cv_^2^ = 0.943 and RMSECV = 75.6), 5-fold Venetian blinds (*R*_cv_^2^ = 0.943 and RMSECV = 75.4), 5-fold continuous blocks (*R*_cv_^2^ = 0.944 and RMSECV = 75.1), the Monte Carlo method, which randomly excludes 20% of molecules with 1000 iterations (*R*_cv_^2^ = 0.942 and RMSECV = 75.4), and bootstrapping with 1000 iterations (*R*_cv_^2^ = 0.942 and RMSECV = 76.4). The absence of change correlation in the model was confirmed by Y-randomization with 1000 iterations, and the average reflected poor regression quality (*R* ^2^ = 0.016 and RMSE = 313.6). Finally, the calibrated model was used to predict the *RIs* of the test set molecules, which demonstrated good predictive ability when the coefficient of prediction (*Q* ^2^ = 0.945) and the root-mean-square error of prediction (RMSEP = 67.4) were analyzed. Thus, the chemoinformatic model is reliable for both modeling and predicting the retention indices of metabolites in mixed flours using the HP-5ms capillary column in GC-QTOF-MS. A comparison of the conformation-independent MLR model developed in this work with similar models reported in Table 1 shows that our QSRR model was built using the largest dataset, ensuring a more generalized chemical domain, and exhibits comparable predictive ability to those based on larger datasets^16,19,23^ or more advanced modeling approaches, such as SVM,^13^ MSOP,^16^ ANN,^21,22,24^ and BRT.^23^

The correlation between the experimental and predicted retention indices of the metabolites is illustrated in Figure 1, and a linear trend that is closely aligned with respect to the 45° reference line is shown, indicating a strong relationship between the experimental and predicted *RI* values. Figure 1b shows the residual distribution along the predicted retention indices, which appear randomly scattered along the zero line, demonstrating the absence of systematic error. Additionally, potential outliers were assessed using a threshold of ±2 times the RMSEC. In this model, two compounds fall outside these limits: 2*H*-pyran-2-one, 6-hexyltetrahydro- and butanal, 3-methyl-. Collectively, Figure 1a,b supports the reliability and precision of the chemoinformatic model described in eq 1, confirming that it aligns with the ordinary least-squares (OLS) assumption for calibrating a predictive quantitative structure-retention relationship.

**Figure 1:**
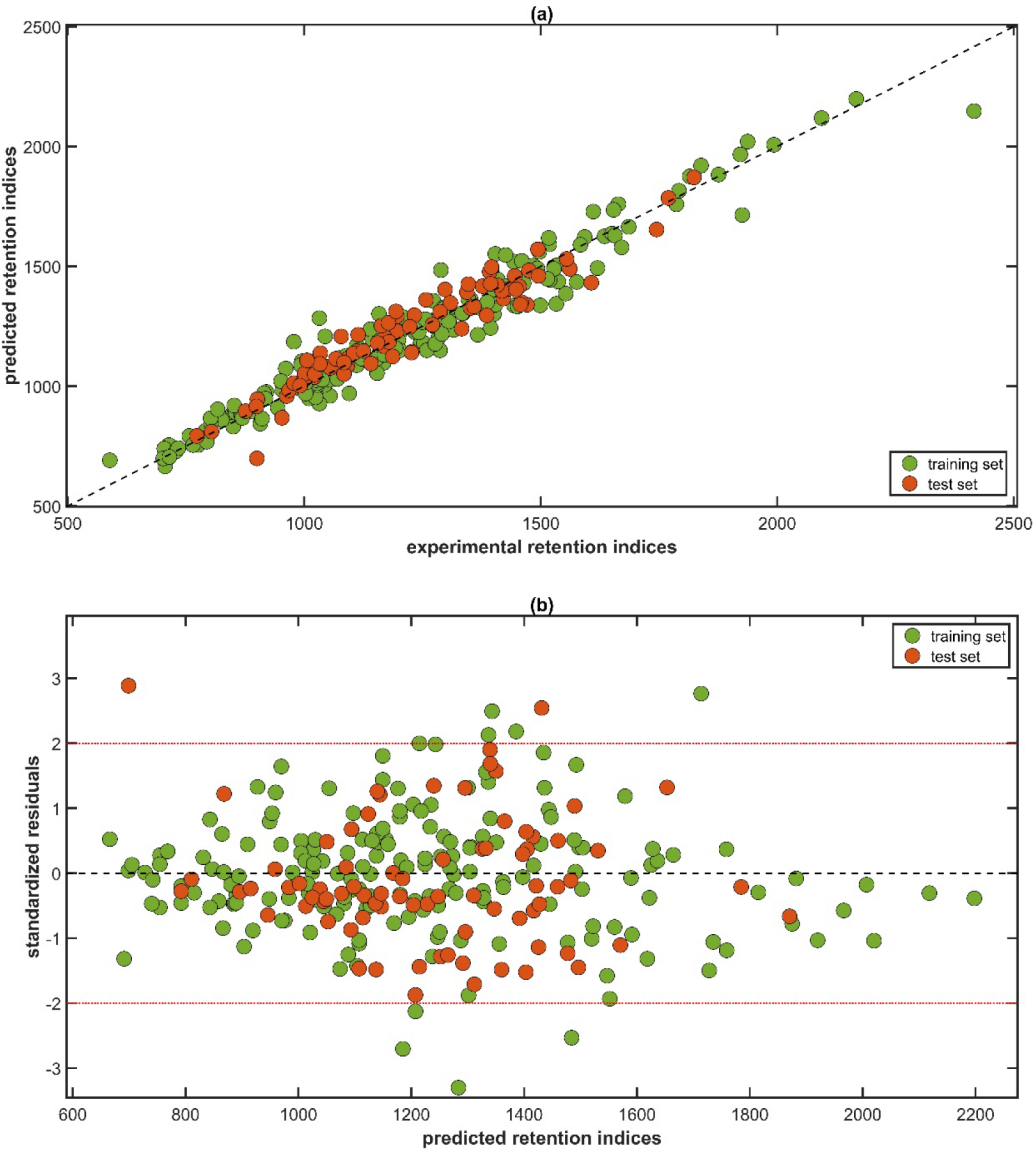
QSRR model to predict the retention index of metabolites identified in coffee and plantain byproduct flours in the HP-5ms capillary column: (a) experimental versus predicted retention indices; and (b) predicted retention indices versus standardized residuals.

#### 3.2.2 Mechanism of Action of the QSRR Model

To analyze the mechanism of action of the three molecular descriptors integrated in the chemoinformatic model of eq 1, the standardized regression coefficients were used. As shown in Figure 2a, the Eta_betaS descriptor (0.756) contributed most significantly to the retention index prediction, followed by MDEC-22 (0.251) and MATS1p (0.205), whereas Figure 2b illustrates the distribution of the *RI* property along the chemical space defined by these molecular features. These three features exhibit a synergistic effect on *RI* prediction; that is, the greater their values, the higher are the corresponding retention indices. This relationship is illustrated in Figure 3, where flavor compounds exhibiting low, intermediate, and high *RIs* are represented.

**Figure 2:**
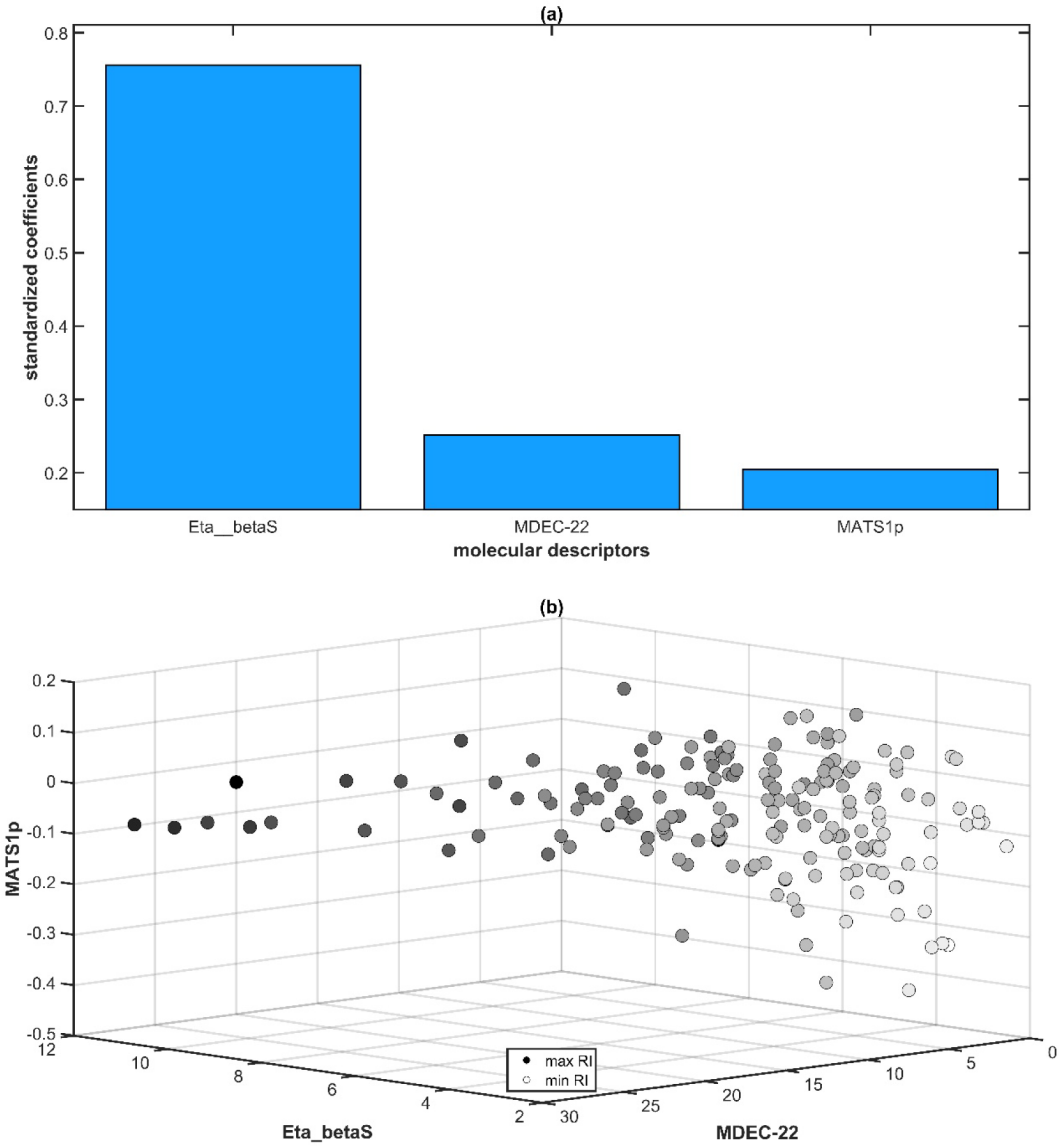
Mechanistic interpretation of the three molecular descriptors included in the chemoinformatic model of eq 1: (a) standardized regression coefficients showing the contribution of each molecular feature; (b) chemical space illustrating the distribution of retention indices along the molecular features.

**Figure 3:**
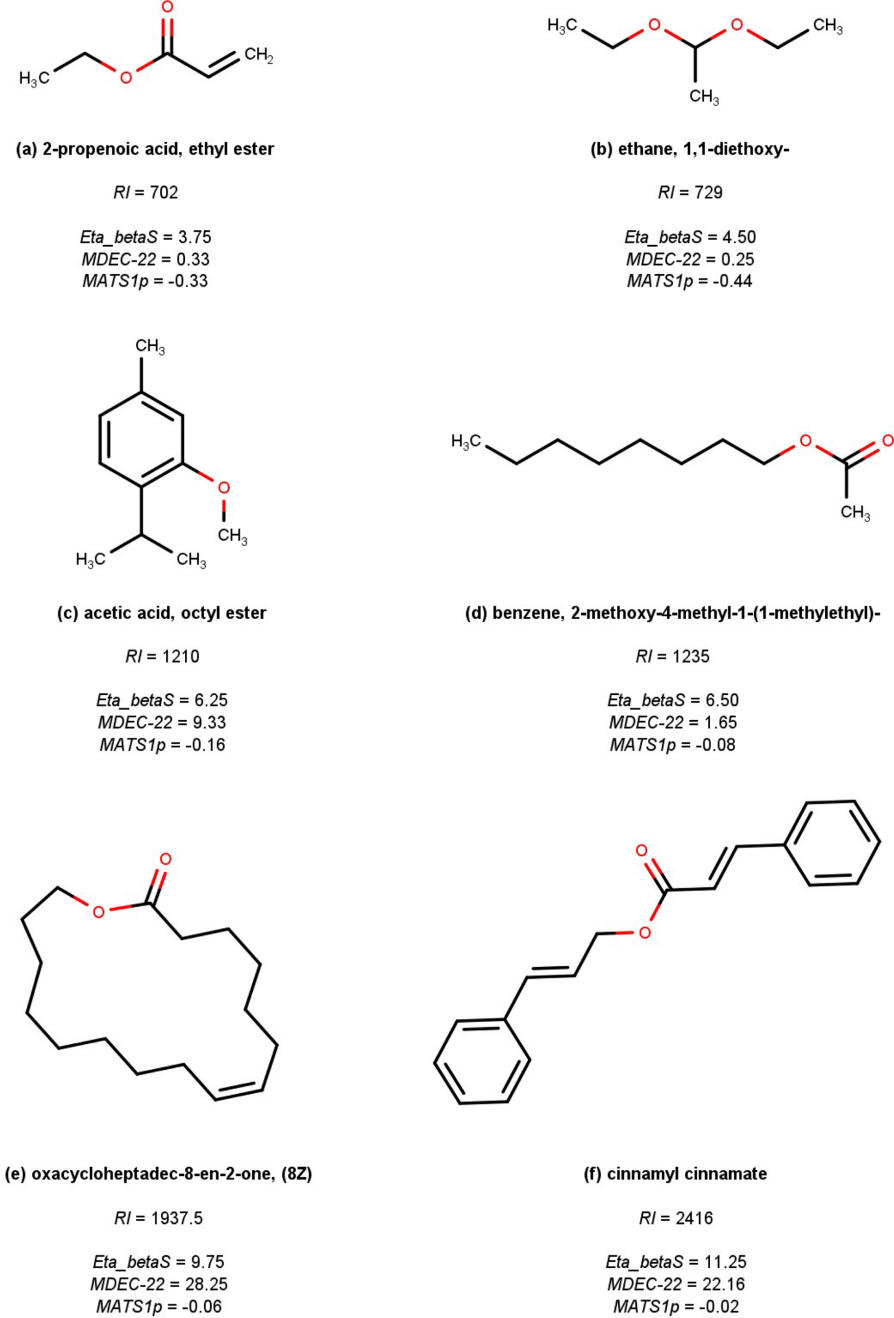
Compounds with low (a,b), medium (c,d), and high (e,f) retention indices quantified in the HP-5ms capillary column by means of gas chromatography–mass spectrometry (GC–MS).

The eta sigma VEM count (Eta_betaS) is an extended topochemical atom (ETA) index that accounts for valence electron mobility (VEM) by considering all sigma bonds in the molecule (except hydrogen bonds). It provides a relative measure of the number of electronegative atoms present in the molecular structure of a compound.^32^ The molecular distance edge (MDE) between all secondary carbons (MDEC-22) is a descriptor based on the geometric means of the topological distances between secondary carbon atoms (CH_2_<) within the molecular graph of flavor compounds. MDE descriptors have been proposed to model and predict the normal boiling point of alkanes.^32^ Finally, the Moran autocorrelation of lag 1 weighted by polarizability (MATS1p) is a two-dimensional autocorrelation descriptor^32^ that characterizes volatile molecules containing atoms with similar polarizability at a lag of 1 within the molecular structure. The synergistic effect of this feature in predicting the *RIs* of volatile organic compounds (VOCs) has been reported elsewhere.^64,65^

#### 3.2.3 Applicability Domain of the QSRR Model

The reliability of a chemoinformatic model in generating new predictions is fundamentally dependent on the assessment of its applicability domain (AD), which defines the theoretical chemical space within which retention index predictions are considered interpolations of the model. The applicability domain of a multiple linear regression (MLR) model is most effectively evaluated by using the Williams plot (Figure 4), where leverage values are plotted against standardized residuals. The threshold for identifying reliable predictions is determined by the warning leverage value (h* = 0.065). Accordingly, the QSRR model described in eq 1 has strong predictive accuracy for the complete test set of molecules.

**Figure 4:**
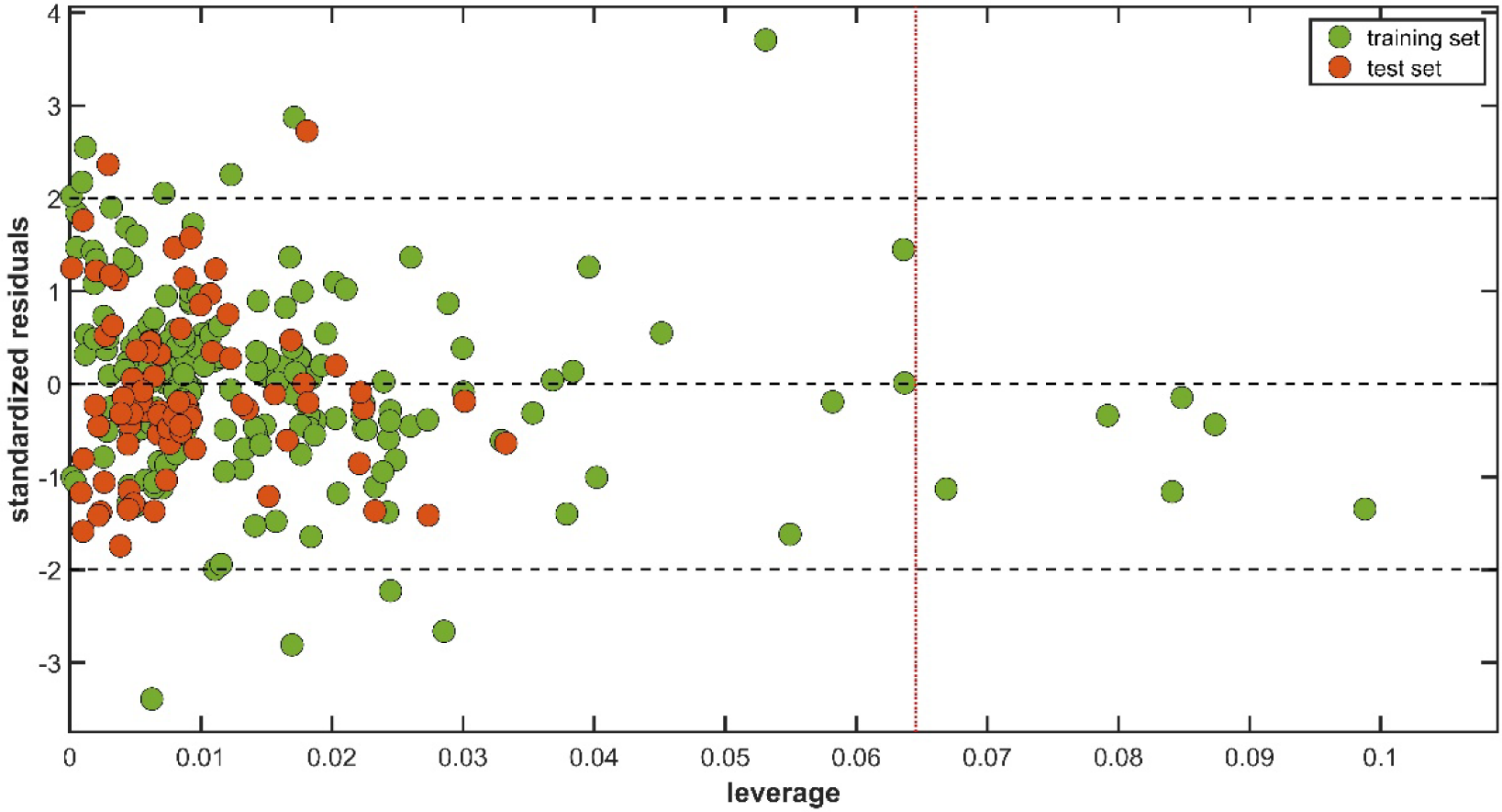
Williams plot for the applicability domain assessment of the QSRR model for the prediction of retention indices of metabolites identified in coffee and plantain byproduct flours in the HP-5ms capillary column. The threshold for reliable predictions is defined by the warning leverage value of h* = 0.065.

#### 3.2.4 Application of the QSRR Model for Retention Index Prediction of Metabolites

The applicability of the QSRR model is related to its ability to predict the retention indices (*RIs*) of new chemical compounds from food matrices analyzed via an HP-5 ms capillary column. To this end, an external dataset comprising the 67 merged metabolites elicited in this work was used. The QSRR model presented in eq 1 was used to predict the *RIs* of these metabolites and to assess the model’s applicability domain (AD). The results showed that 48 compounds fell within the AD, meaning their *RI* predictions were considered reliable, as the leverage values were below the warning threshold (h*). In contrast, 19 molecules fell outside the AD, with leverage values exceeding h*, indicating that their predictions were unreliable due to substantial extrapolation. Table S3 in the Supporting Information provides detailed information on the 67 metabolites, including both experimental and predicted retention indices, the reliability of the predictions derived from the applicability domain assessment, and the values for the three MDs. After excluding the molecules that fell outside the AD, the prediction performance for the external set was *Q* ^2^ = 0.918 and RMSEP = 83.4, confirming the reliability of the QSRR model for new predictions of metabolites derived from coffee and plantain byproduct flours, as well as its potential for generalization to the metabolomic analysis of diverse food matrices and byproduct derivatives by means of GC–MS.

The QSRR model developed in this work offers a powerful and practical tool that enables the identification of metabolites generated by the mechanical processing of food matrices (extrusion, in this case) prior to experimental analysis. It also supports the identification of unknown peaks by comparing predicted *RI* values with experimental data available in open-access libraries. This is particularly useful when reference standards are not available. Furthermore, chemists can examine chemical structures resulting from physical, chemical, or biotechnological processes and use the model to estimate *RIs*, aiding in the identification of unknown compounds. Finally, this model could serve as a starting point for further studies aimed at predicting and transferring retention indices across different chromatographic systems. This approach may facilitate metabolite identification in untargeted analyses of diverse food matrices, thereby enhancing the design, development, and optimization of novel chromatographic methods for the valorization of foods.^66^

## Supplementary Material



## Notes

Multivariate analysis of control and extruded coffee and plantain byproduct flour samples (Figure S1); match scores of the database of 72 metabolites identified in coffee and plantain byproduct flours using gas chromatography–time-of-flight mass spectrometry (GC-MS-QTOF) in the HP-5 ms capillary column (Table S1); details of Yan and coworkers’ database of 265 volatile organic compounds identified using gas chromatography–mass spectrometry (GC–MS) in the HP-5 ms capillary column, which was used to calibrate the QSRR model (Table S2); details of the curated database of metabolites identified in coffee and plantain byproduct flours using gas chromatography–time-of-flight mass spectrometry (GC-MS-QTOF) in the HP-5 ms capillary column, used to predict the retention index by means of the QSRR model (Table S3) (PDF)
