# Supplementary material for: Characterization and Chemoinformatic Prediction of Retention Indices of Metabolites in Coffee and Plantain Byproduct Flours Using Gas Chromatography–Time-of-Flight Mass Spectrometry
Source: J Agric Food Chem. 2025 Nov 17;73(47):30473–87. doi: 10.1021/acs.jafc.5c11135 (PMC12670392; doi:10.1021/acs.jafc.5c11135)
Supplement: Supplementary file 1 [file jf5c11135_si_001.pdf]

Supporting Information

Characterization and Chemoinformatic Prediction of Retention Indices of Metabolites in Coffee and Plantain Byproduct Flours Using Gas Chromatography–Time-of-Flight Mass Spectrometry

Laura Sofía Torres-Valenzuela[a,*], Carolina Franco-Urbano[a], Diana Paola Navia-Porras[b], Nicole Sarmiento[c], Cristian Rojas[c]

[a] Grupo de Investigación GIPAB, Escuela de Ingeniería de Alimentos, Universidad del Valle, Calle 13 No. 100-00, 760032, Cali, Colombia.

[b] Grupo de Investigación Biotecnología, Facultad de Ingeniería, Universidad de San Buenaventura Cali, Carrera 122 # 6-65, Cali 76001, Colombia.

[c] Grupo de Investigación en Quimiometría y QSAR, Facultad de Ciencia y Tecnología, Universidad del Azuay, Av. 24 de Mayo 7-77 y Hernán Malo, Cuenca 010107, Ecuador.

[*]Corresponding author. E-mail: laura.torres@correounivalle.edu.co

Equipment and materials

For byproduct processing, a cutting machine (Poli, Cali, Colombia), forced convection oven ED 115 (Binder, Tuttlingen, Germany), mill M20 (IKA, Königswinter, Germany), and rotap (W.S. TYLER, Mentor, OH, USA) were employed. The extrusion was conducted using a twin-screw extruder (DS32-II, Jainin Saixin Machinery Co®, Jinan, Shandong, China).

A gas chromatograph system 7890B was employed (Agilent Technologies, Santa Clara, California, USA) coupled to a time-of-flight mass selective detector (Agilent Technologies GC/Q-TOF 7250) equipped with a split/splitless injection port (250 °C, divided ratio 50). An Agilent Technologies J&W HP-5ms column (30 m × 0.25 mm, 0.25 μm), with a (5%-phenyl)-methylpolysiloxane was used.

Table S1. Match scores of the database of 72 metabolites identified in coffee and plantain by-product flours using gas chromatography–time-of-flight mass spectrometry (GC-MS-QTOF) in the HP-5ms capillary column.

No.	Metabolite	CAS registry number	Retention index (*RI*)	Match score
1	erythritol	149-32-6	1193	93
2	2,3-butanediol	513-85-9	709	94
3	glycerol	56-81-5	960	90
4	arabitol	488-82-4	1409	67
5	diglycerol	627-82-7	1428	60
6	ethanolamine	141-43-5	952	89
7	glycine	56-40-6	993	65
8	cycloleucine	52-52-8	1046	71
9	glutamic acid	56-86-0	1210	93
10	glucosamine-phosphate	2152-75-2	1492	52
11	galactosamine	90-76-6	1551	50
12	tyramine	51-67-2	1604	56
13	glucosaminic acid	3646-68-2	1685	63
14	valine	72-18-4	764	66
15	valeramide	626-97-1	823	45
16	isoleucine	443-79-8	858	53
17	serine	56-45-1	941	85
18	pipecolic acid	535-75-1	948	66
19	threonine	72-19-5	979	79
20	malonamide	108-13-4	1078	52
21	acetyl-glutamic acid	1188-37-0	1179	47
22	ornithine	70-26-8	1271	50
23	2-deoxy-ribose	533-67-5	1226	52
24	deoxyglucose	154-17-6	1448	56
25	1,5-anhydroglucitol	154-58-5	1187	47
26	methyl-galactopyranoside	3396-99-4	1528	70
27	galactonic acid	576-36-3	1549	72
28	sorbose	87-79-6	1546	53

29	galactose	59-23-4	1551	74
30	mannose	3458-28-4	1551	99
31	sedoheptulose anhydride monohydrate	469-90-9	1599	73
32	glucose	2280-44-6	1608	70
33	glucoheptonic acid	87-74-1	1712	99
34	mucic acid	526-99-8	1717	80
35	1,5-anhydrosorbitol	154-58-5	1511	70
36	xylose	25990-60-7	1313	83
37	ribose	50-69-1	1352	59
38	fructose	57-48-7	1582	94
39	palmitic acid	57-10-3	1722	95
40	nonanoic acid	112-05-0	1039	66
41	heptadecanoic acid	506-12-7	1819	57
42	2-furoic acid	88-14-2	813	71
43	malonic acid	141-82-2	887	95
44	fumaric acid	110-17-8	1025	94
45	hydroxyhexanoic acid	1191-25-9	1083	53
46	malic acid	6915-15-7	1177	91
47	hydroxyglutaric acid	2889-31-8	1263	73
48	aconitic acid	585-84-2	1434	62
49	dihydroxybenzoic acid	99-50-3	1511	52
50	quinic acid	77-95-2	1568	99
51	gluconic acid lactone	90-80-2	1581	67
52	glucuronolactone	32449-92-6	1608	62
53	gallic acid	149-91-7	1653	46
54	shikimic acid	138-59-0	1905	98
55	pyruvic acid	127-17-3	725	87
56	lactic acid	79-33-4	738	98
57	glycolic acid	79-14-1	753	96
58	oxalic acid	144-62-7	812	97
59	hydroxybutyric acid	300-85-6	842	76
60	nicotinic acid	59-67-6	975	82
61	succinic acid	110-15-6	994	98
62	glyceric acid	473-81-4	1017	73

63	dihydroxymalonic acid	560-27-0	1121	55
64	3-hydroxypropanoic acid	503-66-2	1250	47
65	hydroxybenzoic acid	99-96-7	1313	54
66	vanillic acid	121-34-6	1452	71
67	gulonic acid lactone	1128-23-0	1601	70
68	caffeine	58-08-2	1540	97
69	1,2-dihydro-1,2-naphthalenediol	31966-70-8	1313	52
70	4-O-methylphloracetophenone	7507-89-3	1398	49
71	1,3-dihydroxyacetone	96-26-4	926	63
72	phosphoric acid	7664-38-2	960	94

Table S2. Details of Yan and coworkers' database of 265 volatile organic compounds identified using gas chromatography–mass spectrometry (GC-MS) in the HP-5ms capillary column, which was used to calibrate the QSRR model. The Table includes the chemical name, CAS registry number, PubChem CID, SMILES, retention indices, dataset splitting and numerical values of the three conformation-independent molecular descriptors.

No.	Chemical name	CAS number	PubChem CID	SMILES	Retention indices		Dataset splitting	Molecular descriptors		
					Experimental	Predicted		Eta_betaS	MDEC-22	MATS1p
1	benzeneacetic acid, 2-phenylethyl ester	102-20-5	7601	C1=CC=C(C=C1)CCOC(=O)CC2=CC=CC=C2	1922	1966.4	training	10.25	18.27	-0.02
2	acetic acid, octyl ester	112-14-1	8164	CCCCCCCCOC(=O)C	1210	1247.8	training	6.25	9.33	-0.16
3	naphthalene, 2-ethoxy-	93-18-5	7129	CCOC1=CC2=CC=CC=C2C=C1	1528	1497.7	training	7.50	9.93	-0.03
4	benzeneacetic acid, 2-methylpropyl ester	102-13-6	60998	CC(C)COC(=O)CC1=CC=CC=C1	1392	1477.3	test	7.75	8.19	-0.07
5	2-propenal, 3-(2-methoxyphenyl)-	1504-74-1	641298	COC1=CC=CC=C1/C=C/C=O	1533	1343.4	training	6.75	8.36	-0.09
6	octanoic acid, ethyl ester	106-32-1	7799	CCCCCCCC(=O)OCC	1196	1228.8	test	6.25	7.96	-0.16
7	propanoic acid, 2-methyl-, ethyl ester	97-62-1	7342	CCOC(=O)C(C)C	757	792.3	training	4.25	0.00	-0.28
8	2(3H)-furanone, 5-ethyldihydro-	695-06-7	12756	CCC1CCC(=O)O1	1054.5	959.7	training	4.75	1.65	-0.15
9	decanal	112-31-2	8175	CCCCCCCCCC=O	1205	1232.3	training	5.25	13.19	-0.07
10	2-cyclopenten-1-one, 3-methyl-2-(2-pentenyl)-, (Z)-	488-10-8	1549018	CC/C=C\CC1=C(CCC1=O)C	1401	1300.6	training	6.25	5.80	0.00
11	isobutyl acetate	110-19-0	8038	CC(C)COC(=O)C	773	792.3	test	4.25	0.00	-0.28
12	2H-pyran-2-one, tetrahydro-6-nonyl-	2721-22-4	520296	CCCCCCCCCC1CCCC(=O)O1	1926	1713.9	training	8.75	15.64	-0.06
13	oxacycloheptadec-8-en-2-one, (8Z)	123-69-3	5365703	C1CCCCOC(=O)CCCCC/C=C\CCC1	1937.5	2020.1	training	9.75	28.25	-0.06
14	2-buten-1-ol, 3-methyl-	556-82-1	11173	CC(=CCO)C	776	754.8	training	2.75	1.00	-0.06
15	butanoic acid, 3,7-dimethyl-2,6-octadienyl ester, (E)-	106-29-6	5355856	CCCC(=O)OC/C=C(\C)/CCC=C(C)C	1561.5	1489.7	test	8.25	6.24	-0.11
16	2-butanone, 4-(4-hydroxyphenyl)-	5471-51-2	21648	CC(=O)CCC1=CC=C(C=C1)O	1553	1386.1	training	6.50	6.68	0.07
17	2-propen-1-ol, 3-phenyl-	104-54-1	5315892	C1=CC=C(C=C1)/C=C/CO	1306	1304.2	training	5.25	11.47	0.08
18	methyl isovalerate	556-24-1	11160	CC(C)CC(=O)OC	777	792.3	training	4.25	0.00	-0.28
19	cyclohexanol, 5-methyl-2-(1-methylethyl)-, (1α,2α,5α)-	20752-34-5	19244	C[C@@H]1CC[C@@H]([C@@H](C1)O)C(C)C	1174	1206.7	training	5.75	1.65	0.04
20	methyleugenol	93-15-2	7127	COC1=C(C=C(C=C1)CC=C)OC	1404.5	1340.3	training	7.50	4.56	-0.16
21	hexadecanoic acid, ethyl ester	628-97-7	12366	CCCCCCCCCCCCCCCC(=O)OCC	1993	2006.9	training	10.25	24.20	-0.09

22	2-furancarboxaldehyde, 5-methyl-	620-02-0	12097	CC1=CC=C(O1)C=O	963	958.8	test	4.75	1.65	-0.15
23	2H-pyran-2-one, tetrahydro-6-propyl-	698-76-0	12777	CCCC1CCCC(=O)O1	1287	1149.6	training	5.75	4.51	-0.11
24	butanoic acid, ethyl ester	105-54-4	7762	CCCC(=O)OCC	804	810.5	test	4.25	1.31	-0.28
25	butanoic acid, 3-methyl-, phenylmethyl ester	103-38-8	7651	CC(C)CC(=O)OCC1=CC=CC=C1	1396	1477.3	training	7.75	8.19	-0.07
26	2-nonenal, (Z)-	60784-31-8	5354833	CCCCCC/C=C\C=O	1159	1138.5	training	4.75	11.24	-0.08
27	1-butanol, 3-methyl-, propanoate	105-68-0	7772	CCC(=O)OCCC(C)C	968	983.1	test	5.25	1.31	-0.21
28	butanoic acid, 3-methylbutyl ester	106-27-4	7795	CCCC(=O)OCCC(C)C	1055	1076.7	test	5.75	2.41	-0.18
29	acetic acid, 2-phenylethyl ester	103-45-7	7654	CC(=O)OCCC1=CC=CC=C1	1257.5	1360.1	test	6.75	9.56	-0.09
30	propanoic acid, 2-methyl-, phenylmethyl ester	103-28-6	7646	CC(C)C(=O)OCC1=CC=CC=C1	1298	1403.3	test	7.25	7.73	-0.08
31	diphenyl ether	101-84-8	7583	C1=CC=C(C=C1)OC2=CC=CC=C2	1404	1551.9	training	7.50	14.00	-0.03
32	dodecanoic acid, ethyl ester	106-33-2	7800	CCCCCCCCCCCC(=O)OCC	1593	1622.1	training	8.25	15.90	-0.11
33	3-phenylpropanol	122-97-4	31234	C1=CC=C(C=C1)CCCO	1233	1296.1	test	5.25	11.47	0.07
34	undecanal	112-44-7	8186	CCCCCCCCCCC=O	1306	1327.1	training	5.75	15.16	-0.06
35	1-cyclohexene-1-methanol, 4-(1-methylethenyl)-	536-59-4	10819	CC(=C)C1CCC(=CC1)CO	1301	1257.9	training	5.75	4.88	0.05
36	2-naphthyl methyl ketone	93-08-3	7122	CC(=O)C1=CC2=CC=CC=C2C=C1	1620	1492.5	training	7.25	8.78	0.04
37	cinnamyl cinnamate	122-69-0	1550890	C1=CC=C(C=C1)/C=C/COC(=O)/C=C/C2=CC=CC=C2	2416	2147.5	training	11.25	22.16	-0.02
38	acetic acid, 4-methylphenyl ester	140-39-6	8797	CC1=CC=C(C=C1)OC(=O)C	1170	1203.7	test	6.25	3.30	-0.10
39	benzenemethanol, 4-methoxy-, acetate	104-21-2	7695	CC(=O)OCC1=CC=C(C=C1)OC	1421	1365.6	test	7.75	4.88	-0.18
40	butanoic acid, 2-methyl-, ethyl ester	7452-79-1	24020	CCC(C)C(=O)OCC	849	885.6	training	4.75	0.25	-0.24
41	2-octanone	111-13-7	8093	CCCCCCC(=O)C	991	990.0	training	4.25	5.68	-0.09
42	acetoin	513-86-0	179	CC(C(=O)C)O	714	754.5	training	3.00	0.00	-0.09
43	2H-pyran-2-one, 6-hexyltetrahydro-	710-04-3	61204	CCCCCCC1CCCC(=O)O1	1607	1431.2	test	7.25	9.74	-0.08
44	1-butanol, 3-methyl-, formate	110-45-2	8052	CC(C)CCOC=O	792	815.2	training	4.25	1.65	-0.28
45	2-hexenal, (E)-	6728-26-3	5281168	CCC/C=C/C=O	852	847.1	training	3.25	5.68	-0.11
46	cedrol	77-53-2	65575	C[C@@H]1CC[C@@H]2[C@]13CC[C@@]([C@H](C3)C2(C)C)(C)O	1611	1728.2	training	9.25	4.08	0.11
47	hexanoic acid, 2-propenyl ester	123-68-2	31266	CCCCCC(=O)OCC=C	1079	1120.5	training	5.75	5.48	-0.18

48	octanoic acid, 3-methylbutyl ester	2035-99-6	16255	CCCCCCCC(=O)OCCC(C)C	1445	1459.4	test	7.75	9.13	-0.12
49	benzoic acid	65-85-0	243	C1=CC=C(C=C1)C(=O)O	1178	1221.5	training	5.00	6.08	0.12
50	β-phenylethyl butyrate	103-52-6	7658	CCCC(=O)OCCC1=CC=CC=C1	1443	1520.0	training	7.75	11.27	-0.07
51	butanoic acid, butyl ester	109-21-7	7983	CCCCOC(=O)CCC	995	1018.1	training	5.25	3.84	-0.21
52	benzophenone	119-61-9	3102	C1=CC=C(C=C1)C(=O)C2=CC=CC=C2	1635	1625.3	training	7.75	14.00	0.03
53	2-nonanone	821-55-6	13187	CCCCCCCC(=O)C	1091	1084.8	test	4.75	7.47	-0.08
54	propanoic acid, 2-methyl-, octyl ester	109-15-9	61024	CCCCCCCCOC(=O)C(C)C	1344	1392.1	test	7.25	9.33	-0.13
55	butanoic acid, 3-methyl-, ethyl ester	108-64-5	7945	CCOC(=O)CC(C)C	852	886.7	training	4.75	0.33	-0.24
56	piperonal	120-57-0	8438	C1OC2=C(O1)C=C(C=C2)C=O	1336	1304.9	training	7.25	3.88	-0.15
57	triethyl citrate	77-93-0	6506	CCOC(=O)CC(CC(=O)OCC)(C(=O)OCC)O	1664	1759.1	training	11.50	2.26	-0.25
58	2-furanmethanol, acetate	623-17-6	12170	CC(=O)OCC1=CC=CO1	995	1104.2	training	6.25	3.30	-0.26
59	formic acid, phenylmethyl ester	104-57-4	7708	C1=CC=C(C=C1)COC=O	1078	1207.5	test	5.75	8.74	-0.11
60	octanoic acid	124-07-2	379	CCCCCCCC(=O)O	1179	1142.8	training	5.00	7.47	-0.04
61	D-carvone	2244-16-8	16724	CC1=CC[C@@H](CC1=O)C(=C)C	1246	1179.8	training	5.75	1.65	0.00
62	naphthalene, 2-methoxy-	93-04-9	7119	COC1=CC2=CC=CC=C2C=C1	1454.5	1415.7	test	7.00	8.78	-0.03
63	2-nonen-1-ol, (E)-	31502-14-4	5364941	CCCCCC/C=C/CO	1168	1167.7	test	4.75	11.24	-0.03
64	butanoic acid, 3-methyl-	503-74-2	10430	CC(C)CC(=O)O	850	831.4	training	3.50	0.00	-0.06
65	benzaldehyde	100-52-7	240	C1=CC=C(C=C1)C=O	961	1073.9	training	4.25	7.73	0.00
66	2-hexen-1-ol, (E)-	928-95-0	5318042	CCC/C=C/CO	865	889.9	training	3.25	5.68	-0.05
67	triacetin	102-76-1	5541	CC(=O)OCC(COC(=O)C)OC(=O)C	1352	1362.5	training	9.25	0.50	-0.39
68	benzoic acid, 2-hydroxy-, ethyl ester	118-61-6	8365	CCOC(=O)C1=CC=CC=C1O	1273	1356.1	training	7.00	4.11	-0.03
69	acetic acid, heptyl ester	112-06-1	8159	CCCCCCCOC(=O)C	1111	1146.8	test	5.75	7.47	-0.18
70	butanoic acid, 3-methyl-, 3-phenyl-2-propenyl ester	140-27-2	5355855	CC(C)CC(=O)OC/C=C/C1=CC=CC=C1	1686	1664.5	training	8.75	12.07	-0.06
71	2(3H)-furanone, 5-hexyldihydro-	706-14-9	12813	CCCCCCC1CCC(=O)O1	1471	1339.8	test	6.75	8.07	-0.09
72	allyl nonanoate	7493-72-3	61410	CCCCCCCCC(=O)OCC=C	1377	1416.9	test	7.25	11.07	-0.13
73	isoamyl cinnamate	7779-65-9	5273467	CC(C)CCOC(=O)/C=C/C1=CC=CC=C1	1745	1653.4	test	8.75	11.27	-0.06
74	1-butanol, 3-methyl-, acetate	123-92-2	31276	CC(C)CCOC(=O)C	876	896.0	test	4.75	1.00	-0.24

75	1-decanol	112-30-1	8174	CCCCCCCCCCO	1271	1256.4	test	5.25	13.19	-0.03
76	ethanone, 1-(2,4-dimethylphenyl)-	89-74-7	6985	CC1=CC(=C(C=C1)C(=O)C)C	1253	1179.8	training	5.75	1.65	0.00
77	2H-pyran-2-one, tetrahydro-6-methyl-	823-22-3	13204	CC1CCCC(=O)O1	1095	969.9	training	4.75	2.38	-0.15
78	butanoic acid, phenylmethyl ester	103-37-7	7650	CCCC(=O)OCC1=CC=CC=C1	1347	1425.6	test	7.25	9.34	-0.08
79	3-hexen-1-ol	544-12-7	10993	CCC=CCCO	855	889.9	training	3.25	5.68	-0.05
80	2-hexen-1-ol, acetate, (E)-	2497-18-9	2733294	CCC/C=C/COC(=O)C	1015	1045.1	test	5.25	5.68	-0.20
81	decanedioic acid, diethyl ester	110-40-7	8049	CCOC(=O)CCCCCCCCC(=O)OCC	1787	1758.6	training	10.00	12.70	-0.18
82	benzoic acid, 2-hydroxy-, 2-methylpropyl ester	87-19-4	6873	CC(C)COC(=O)C1=CC=CC=C1O	1475	1482.9	test	8.00	4.11	-0.03
83	1-hexanol	111-27-3	8103	CCCCCCO	867	880.5	training	3.25	5.68	-0.06
84	2(5H)-furanone, 5-ethyl-3-hydroxy-4-methyl-	698-10-2	61199	CCC1C(=C(C(=O)O1)O)C	1195	1155.0	training	6.00	0.00	-0.05
85	tetradecanoic acid, ethyl ester	124-06-1	31283	CCCCCCCCCCCCCC(=O)OCC	1792.3	1815.1	training	9.25	20.02	-0.10
86	benzenemethanol, 4-methoxy-	105-13-5	7738	COC1=CC=C(C=C1)CO	1284	1203.6	training	5.75	4.88	-0.03
87	2-heptanone	110-43-0	8051	CCCCCC(=O)C	891	894.6	training	3.75	3.97	-0.10
88	n-decanoic acid	334-48-5	2969	CCCCCCCCCC(=O)O	1370	1326.9	training	6.00	11.24	-0.03
89	pentanoic acid, butyl ester	591-68-4	61137	CCCCC(=O)OCCCC	1093	1116.7	test	5.75	5.30	-0.18
90	ethyl vanillin	121-32-4	8467	CCOC1=C(C=CC(=C1)C=O)O	1459	1350.1	test	7.00	3.69	-0.03
91	benzyl benzoate	120-51-4	2345	C1=CC=C(C=C1)COC(=O)C2=CC=CC=C2	1770	1784.8	test	9.25	14.55	-0.03
92	methyl salicylate	119-36-8	4133	COC(=O)C1=CC=CC=C1O	1196	1291.7	test	6.50	3.97	-0.02
93	pentyl octanoate	638-25-5	61185	CCCCCCCC(=O)OCCCCC	1484	1502.7	training	7.75	12.26	-0.12
94	3-hexen-1-ol, acetate, (Z)-	3681-71-8	5363388	CC/C=C\CCOC(=O)C	1006	1045.1	training	5.25	5.68	-0.20
95	benzeneethanol, α,α-dimethyl-	100-86-7	7531	CC(C)(CC1=CC=CC=C1)O	1158	1301.8	training	5.75	7.73	0.06
96	isopropyl myristate	110-27-0	8042	CCCCCCCCCCCCCC(=O)OC(C)C	1824	1870.6	test	9.75	19.18	-0.09
97	propanoic acid, 2-methyl-, 2-phenylethyl ester	103-48-0	7655	CC(C)C(=O)OCCC1=CC=CC=C1	1396	1496.3	test	7.75	9.56	-0.07
98	2-tridecanone	593-08-8	11622	CCCCCCCCCCCC(=O)C	1495	1460.2	test	6.75	15.16	-0.05
99	benzeneacetic acid, phenylmethyl ester	102-16-9	60999	C1=CC=C(C=C1)CC(=O)OCC2=CC=CC=C2	1815	1875.0	training	9.75	16.35	-0.02
100	acetic acid, decyl ester	112-17-4	8167	CCCCCCCCCCOC(=O)C	1409	1445.4	training	7.25	13.19	-0.13
101	butanoic acid, 3-methyl-, 3-methylbutyl ester	659-70-1	12613	CC(C)CCOC(=O)CC(C)C	1104.5	1136.7	test	6.25	1.31	-0.16

102	2-undecanone	112-12-9	8163	CCCCCCCCCC(=O)C	1293	1272.9	training	5.75	11.24	-0.06
103	benzoic acid, 2-hydroxy-, phenylmethyl ester	118-58-1	8363	C1=CC=C(C=C1)COC(=O)C2=CC=CC=C2O	1876	1882.3	training	10.00	11.93	0.03
104	4H-pyran-4-one, 2-ethyl-3-hydroxy-	4940-11-8	21059	CCC1=C(C(=O)C=CO1)O	1197	1181.8	training	6.00	1.31	-0.04
105	2(3H)-furanone, 5-methyl-	591-12-8	11559	CC1=CCC(=O)O1	869	867.7	training	4.25	1.00	-0.18
106	2(3H)-furanone, dihydro-5-methyl-	108-29-2	7921	CC1CCC(=O)O1	953	868.4	test	4.25	1.00	-0.18
107	benzene, 1-methoxy-4-methyl-	104-93-8	7731	CC1=CC=C(C=C1)OC	1021	1038.0	test	5.00	3.30	-0.11
108	p-cymene	99-87-6	7463	CC1=CC=C(C=C1)C(C)C	1025	1107.6	training	5.00	3.30	0.00
109	heptanoic acid, ethyl ester	106-30-9	7797	CCCCCCC(=O)OCC	1127	1127.8	training	5.75	6.10	-0.18
110	butanedioic acid, dimethyl ester	106-65-0	7820	COC(=O)CCC(=O)OC	1032	927.7	training	6.00	1.00	-0.44
111	2(3H)-furanone, 5-butyldihydro-	104-50-7	7704	CCCCC1CCC(=O)O1	1259	1149.6	training	5.75	4.51	-0.11
112	δ-nonalactone	3301-94-8	18698	CCCCC1CCCC(=O)O1	1394	1243.1	training	6.25	6.12	-0.10
113	benzaldehyde, 4-ethoxy-	10031-82-0	24834	CCOC1=CC=C(C=C1)C=O	1333	1240.1	test	6.25	5.93	-0.10
114	2(4H)-benzofuranone, 5,6,7,7a-tetrahydro-4,4,7a-trimethyl-	15356-74-8	27209	CC1(CCCC2(C1=CC(=O)O2)C)C	1537	1436.3	training	7.75	2.94	-0.02
115	benzene, 1,3-dimethoxy-	151-10-0	9025	COC1=CC(=CC=C1)OC	1168	1097.4	training	6.00	3.53	-0.22
116	nonanoic acid, ethyl ester	123-29-5	31251	CCCCCCCCC(=O)OCC	1295	1328.5	training	6.75	9.89	-0.15
117	octanoic acid, methyl ester	111-11-5	8091	CCCCCCCC(=O)OC	1125	1146.8	test	5.75	7.47	-0.18
118	benzoic acid, ethyl ester	93-89-0	7165	CCOC(=O)C1=CC=CC=C1	1172	1246.7	training	6.25	6.41	-0.10
119	α-ionone	127-41-3	5282108	CC1=CCCC(C1/C=C/C(=O)C)(C)C	1431	1405.3	test	7.25	4.21	0.00
120	2H-pyran-2-one, tetrahydro-6-pentyl-	705-86-2	12810	CCCCCC1CCCC(=O)O1	1499	1337.1	training	6.75	7.88	-0.09
121	heptanoic acid, 3-methylbutyl ester	109-25-1	66955	CCCCCCC(=O)OCCC(C)C	1347	1363.4	training	7.25	7.26	-0.13
122	9,12-octadecadienoic acid (Z,Z)-, methyl ester	112-63-0	5284421	CCCCC/C=C\C/C=C\CCCCCCCC(=O)OC	2094	2118.3	training	10.75	27.41	-0.08
123	menthyl isovalerate	16409-46-4	565690	CC1CCC(C(C1)OC(=O)CC(C)C)C(C)C	1518	1591.3	training	9.25	2.00	-0.06
124	benzene, 2-methoxy-4-methyl-1-(1-methylethyl)-	1076-56-8	14104	CC1=CC(=C(C=C1)C(C)C)OC	1235	1224.4	training	6.50	1.65	-0.08
125	benzeneacetaldehyde, α-ethylidene-	4411-89-6	20446	CC=C(C=O)C1=CC=CC=C1	1274	1275.6	training	5.75	8.57	0.00
126	hexanoic acid, 2-methyl-	4536-23-6	20653	CCCCC(C)C(=O)O	1043	1005.0	training	4.50	2.38	-0.04
127	phenol, 2-(1-methylethyl)-	88-69-7	6943	CC(C)C1=CC=CC=C1O	1199	1192.2	training	5.25	3.97	0.07
128	1-hexanol, 3,5,5-trimethyl-	3452-97-9	18938	CC(CCO)CC(C)(C)C	1047	1030.6	training	4.75	1.65	-0.04

129	benzenepropanal	104-53-0	7707	C1=CC=C(C=C1)CCC=O	1163	1252.3	test	5.25	11.47	0.00
130	2(3H)-furanone, 5-heptyldihydro-	104-67-6	7714	CCCCCCCC1CCC(=O)O1	1576	1434.6	training	7.25	9.98	-0.08
131	formic acid, pentyl ester	638-49-3	12529	CCCCCOC=O	826	859.2	training	4.25	4.83	-0.28
132	3-hexen-1-ol, formate, (Z)-	33467-73-1	5365587	CC/C=C\CCOC=O	920	975.8	training	4.75	6.63	-0.23
133	phenol, 2-methoxy-	90-05-1	460	COC1=CC=CC=C1O	1090	1127.7	training	5.25	3.97	-0.03
134	2-propanone, 1-(4-methoxyphenyl)-	122-84-9	31231	CC(=O)CC1=CC=C(C=C1)OC	1386	1295.4	test	6.75	4.88	-0.09
135	pyrazine, 3-ethyl-2,5-dimethyl-	13360-65-1	25916	CCC1=NC(=CN=C1C)C	1078	1097.1	training	6.00	0.25	-0.15
136	benzaldehyde, 4-ethyl-	4748-78-1	20861	CCC1=CC=C(C=C1)C=O	1179	1184.7	test	5.25	6.58	0.00
137	caryophyllene	87-44-5	5281515	C/C/1=C\CCC(=C)[C@H]2CC([C@@H]2CC1)(C)C	1426	1547.1	training	8.00	5.77	0.04
138	benzene, 1,1’-[oxybis(methylene)]bis-	103-50-4	7657	C1=CC=C(C=C1)COCC2=CC=CC=C2	1654	1735.4	training	8.50	17.77	-0.03
139	nonanoic acid, methyl ester	1731-84-6	15606	CCCCCCCCC(=O)OC	1223	1247.8	test	6.25	9.33	-0.16
140	pentanoic acid, ethyl ester	539-82-2	10882	CCCCC(=O)OCC	852	919.5	training	4.75	2.70	-0.24
141	isopropyl acetate	108-21-4	7915	CC(C)OC(=O)C	589	691.2	training	3.75	0.00	-0.34
142	humulene	6753-98-6	5281520	C/C/1=C\CC(/C=C/C/C(=C/CC1)/C)(C)C	1460	1522.3	training	7.50	10.37	0.00
143	2,4-decadienal, (E,E)-	25152-84-5	5283349	CCCCC/C=C/C=C/C=O	1316	1235.2	training	5.25	13.19	-0.07
144	methional	3268-49-3	18635	CSCCC=O	907	843.2	training	2.75	2.38	0.05
145	2,6,6-trimethyl-2-cyclohexene-1,4-dione	1125-21-9	62374	CC1=CC(=O)CC(C1=O)(C)C	1144	1195.6	training	6.00	0.50	0.00
146	5-methyl-2-phenyl-2-hexenal	21834-92-4	5370602	CC(C)C/C=C(/C=O)\C1=CC=CC=C1	1493	1491.2	training	7.25	10.41	0.00
147	oxiranecarboxylic acid, 3-methyl-3-phenyl-, ethyl ester	77-83-8	6501	CCOC(=O)C1C(O1)(C)C2=CC=CC=C2	1517	1618.4	training	9.25	5.70	-0.10
148	2-octenal, (E)-	2548-87-0	5283324	CCCCC/C=C/C=O	1057	1042.7	training	4.25	9.33	-0.09
149	benzenemethanol, 4-methoxy-, formate	122-91-8	61054	COC1=CC=C(C=C1)COC=O	1334	1304.1	training	7.25	5.96	-0.20
150	nonanal	124-19-6	31289	CCCCCCCCC=O	1104	1136.9	training	4.75	11.24	-0.08
151	butanal, 3-methyl-	590-86-3	11552	CC(C)CC=O	900	699.0	test	2.75	1.00	-0.15
152	cinnamaldehyde, (E)-	14371-10-9	637511	C1=CC=C(C=C1)/C=C/C=O	1272	1252.3	training	5.25	11.47	0.00
153	benzaldehyde, 4-(1-methylethyl)-	122-03-2	326	CC(C)C1=CC=C(C=C1)C=O	1242	1224.6	training	5.75	4.88	0.00
154	octanal, 7-hydroxy-3,7-dimethyl-	107-75-5	7888	CC(CCCC(C)(C)O)CC=O	1288	1233.8	training	6.00	4.51	-0.03
155	6-octenal, 3,7-dimethyl-, (R)-	2385-77-5	75427	C[C@H](CCC=C(C)C)CC=O	1153	1113.6	training	5.25	4.51	-0.07

156	cinnamaldehyde, α-pentyl-	122-40-7	1712058	CCCCC/C(=C/C1=CC=CC=C1)/C=O	1651	1636.4	training	7.75	16.32	0.00
157	2-buten-1-one, 1-(2,6,6-trimethyl-1-cyclohexen-1-yl)-	35044-68-9	32052	CC=CC(=O)C1=C(CCCC1(C)C)C	1418	1397.6	test	7.25	3.65	0.00
158	2-buten-1-one, 1-(2,6,6-trimethyl-2-cyclohexen-1-yl)-, (E)-	24720-09-0	5366077	C/C=C/C(=O)C1C(=CCCC1(C)C)C	1393	1397.6	training	7.25	3.65	0.00
159	2,4-heptadienal, (E,E)-	4313-03-5	5283321	CC/C=C/C=C/C=O	1010	949.1	training	3.75	7.47	-0.09
160	cyclohexanone, 2-(1-mercapto-1-methylethyl)-5-methyl-	38462-22-5	61982	CC1CCC(C(=O)C1)C(C)(C)S	1367	1214.5	training	6.25	1.65	-0.05
161	α-irone	79-69-6	5371002	CC1CC=C(C(C1(C)C)/C=C/C(=O)C)C	1519	1443.8	training	7.75	2.41	0.00
162	5,9-undecadien-2-one, 6,10-dimethyl-	3796-70-1	1549778	CC(=CCC/C(=C/CCC(=O)C)/C)C	1443.5	1336.8	training	6.75	6.12	-0.05
163	10-undecenal	112-45-8	8187	C=CCCCCCCCCC=O	1299	1328.2	training	5.75	15.16	-0.06
164	pentanoic acid, 3-methyl-	105-43-1	7755	CCC(C)CC(=O)O	944	910.0	training	4.00	0.50	-0.05
165	ethanone, 1-(4-methylphenyl)-	122-00-9	8500	CC1=CC=C(C=C1)C(=O)C	1186	1139.3	training	5.25	3.30	0.00
166	benzaldehyde, 2-hydroxy-	90-02-8	6998	C1=CC=C(C(=C1)C=O)O	1044	1207.7	training	5.00	5.09	0.12
167	5-heptenal, 2,6-dimethyl-	106-72-9	61016	CC(CCC=C(C)C)C=O	1054	1026.5	training	4.75	3.15	-0.08
168	dodecanal	112-54-9	8194	CCCCCCCCCCCC=O	1408	1421.6	test	6.25	17.16	-0.06
169	4-heptenal, (Z)-	6728-31-0	5362814	CC/C=C\CCC=O	901	945.8	test	3.75	7.47	-0.10
170	cyclohexanone, 5-methyl-2-(1-methylethyl)-, (2R-cis)-	1196-31-2	70962	C[C@@H]1CC[C@@H](C(=O)C1)C(C)C	1155	1179.8	test	5.75	1.65	0.00
171	acetophenone	98-86-2	7410	CC(=O)C1=CC=CC=C1	1067	1114.4	test	4.75	6.08	0.00
172	hexanal	66-25-1	6184	CCCCCC=O	803	843.5	training	3.25	5.68	-0.12
173	pentanal	110-62-3	8063	CCCCC=O	704	740.0	training	2.75	3.97	-0.15
174	3-(4-isopropylphenyl)-2-methylpropionaldehyde	103-95-7	517827	CC(C)C1=CC=C(C=C1)CC(C)C=O	1464	1429.5	training	7.25	5.96	0.00
175	ethane, 1,1-diethoxy-	105-57-7	7765	CCOC(C)OCC	729	728.2	training	4.50	0.25	-0.44
176	1-octen-3-ol	3391-86-4	18827	CCCCCC(C=C)O	978	1013.1	test	4.25	4.83	-0.03
177	1-octanol	111-87-5	957	CCCCCCCCO	1069.3	1069.8	training	4.25	9.33	-0.04
178	acetic acid, phenyl-, isopentyl ester	102-19-2	7600	CC(C)CCOC(=O)CC1=CC=CC=C1	1497	1560.3	training	8.25	9.34	-0.07
179	2-propen-1-ol, 3-phenyl-, propanoate	103-56-0	5355850	CCC(=O)OC/C=C/C1=CC=CC=C1	1555	1531.0	test	7.75	12.07	-0.07
180	6-octen-1-ol, 3,7-dimethyl-, propanoate	141-14-0	8834	CCC(=O)OCCC(C)CCC=C(C)C	1448	1403.9	test	7.75	5.07	-0.12
181	2,6-octadien-1-ol, 3,7-dimethyl-, formate, (E)-	105-86-2	5282109	CC(=CCC/C(=C/COC=O)/C)C	1306	1269.4	training	6.75	5.48	-0.14
182	3-(methylthio)propanoic acid methyl ester	13532-18-8	61641	COC(=O)CCSC	1024	953.6	training	4.25	1.00	-0.04

183	benzene, ethoxy-	103-73-1	7674	CCOC1=CC=CC=C1	993	1088.5	training	5.00	6.95	-0.11
184	2-methylheptanoic acid	1188-02-9	14475	CCCCCC(C)C(=O)O	1141	1094.2	test	5.00	3.97	-0.04
185	1-hexanol, 2-ethyl-	104-76-7	7720	CCCCC(CC)CO	1028	1004.5	training	4.25	4.61	-0.04
186	3,4-hexanedione	4437-51-8	62539	CCC(=O)C(=O)CC	802	866.5	training	4.00	0.33	-0.12
187	3-hexenoic acid, (E)-	1577-18-0	5282708	CC/C=C/CC(=O)O	1003	969.2	training	4.00	3.97	-0.03
188	propanoic acid	79-09-4	1032	CCC(=O)O	706	665.8	training	2.50	0.00	-0.13
189	heptanoic acid	111-14-8	8094	CCCCCCC(=O)O	1084	1050.6	test	4.50	5.68	-0.04
190	2H-pyran, tetrahydro-4-methyl-2-(2-methyl-1-propenyl)-	16409-43-1	27866	CC1CCOC(C1)C=C(C)C	1111	1169.5	training	6.00	2.62	-0.09
191	propanoic acid, 2-methyl-	79-31-2	6590	CC(C)C(=O)O	765	754.5	training	3.00	0.00	-0.09
192	2,6-octadien-1-ol, 3,7-dimethyl-, (Z)-	106-25-2	643820	CC(=CCC/C(=C\CO)/C)C	1228	1144.3	test	5.25	4.51	-0.02
193	1,6,10-dodecatrien-3-ol, 3,7,11-trimethyl-	7212-44-4	5284507	CC(=CCC/C(=C/CCC(C)(C=C)O)/C)C	1534	1504.1	training	7.75	7.21	-0.01
194	1-butanol, 3-methyl-	123-51-3	31260	CC(C)CCO	734	741.8	training	2.75	1.00	-0.08
195	benzyl alcohol	100-51-6	244	C1=CC=C(C=C1)CO	1034	1137.8	test	4.25	7.73	0.10
196	hexanoic acid, ethyl ester	123-66-0	31265	CCCCCC(=O)OCC	999	1024.9	test	5.25	4.33	-0.21
197	acetic acid, nonyl ester	143-13-5	8918	CCCCCCCCCOC(=O)C	1309	1347.2	test	6.75	11.24	-0.15
198	6-octen-1-ol, 3,7-dimethyl-, acetate	150-84-5	9017	CC(CCC=C(C)C)CCOC(=O)C	1352	1326.1	test	7.25	4.51	-0.13
199	butanoic acid, 3-methyl-, 2-phenylethyl ester	140-26-1	8792	CC(C)CC(=O)OCCC1=CC=CC=C1	1494	1570.7	test	8.25	10.10	-0.07
200	ethyl oleate	111-62-6	5363269	CCCCCCCC/C=C\CCCCCCCC(=O)OCC	2167	2198.0	training	11.25	28.42	-0.08
201	citronellol	106-22-9	8842	CC(CCC=C(C)C)CCO	1227	1140.0	test	5.25	4.51	-0.03
202	2-octynoic acid, methyl ester	111-12-6	8092	CCCCCC#CC(=O)OC	1202	1149.6	training	5.75	7.47	-0.18
203	propanedioic acid, diethyl ester	105-53-3	7761	CCOC(=O)CC(=O)OCC	1070	1030.0	training	6.50	0.79	-0.37
204	butanoic acid, propyl ester	105-66-8	7770	CCCC(=O)OCCC	899	915.4	test	4.75	2.41	-0.24
205	citronellyl butyrate	141-16-2	8835	CCCC(=O)OCCC(C)CCC=C(C)C	1528	1489.0	training	8.25	6.24	-0.11
206	butanoic acid, 2-methylpropyl ester	539-90-2	10885	CCCC(=O)OCC(C)C	954	983.1	training	5.25	1.31	-0.21
207	cyclopentaneacetic acid, 3-oxo-2-pentyl-, methyl ester	24851-98-7	102861	CCCCCC1C(CCC1=O)CC(=O)OC	1657	1628.2	training	9.00	7.39	-0.07
208	isopentyl hexanoate	2198-61-0	16617	CCCCCC(=O)OCCC(C)C	1249	1267.4	training	6.75	5.48	-0.15
209	6-octen-1-ol, 3,7-dimethyl-, formate	105-85-1	7778	CC(CCC=C(C)C)CCOC=O	1275	1268.3	training	6.75	5.48	-0.15

210	1,6-octadien-3-ol, 3,7-dimethyl-	78-70-6	6549	CC(=CCCC(C)(C=C)O)C	1099	1125.5	training	5.25	3.15	-0.02
211	isobornyl acetate	125-12-2	637531	CC(=O)O[C@@H]1C[C@H]2CC[C@@]1(C2(C)C)C	1289	1484.4	training	8.25	1.65	-0.02
212	propanoic acid, 2-methyl-, 3-phenyl-2-propenyl ester	103-59-3	5355851	CC(C)C(=O)OC/C=C/C1=CC=CC=C1	1584	1589.6	training	8.25	11.47	-0.07
213	benzeneacetic acid, methyl ester	101-41-7	7559	COC(=O)CC1=CC=CC=C1	1178	1265.0	test	6.25	7.73	-0.10
214	2-propenoic acid, ethyl ester	140-88-5	8821	CCOC(=O)C=C	702	698.8	training	3.75	0.33	-0.33
215	propanethioic acid, S-(2-furanylmethyl) ester	59020-85-8	62143	CCC(=O)SCC1=CC=CO1	1256	1279.3	training	6.25	3.73	0.01
216	n-propyl acetate	109-60-4	7997	CCCOC(=O)C	715	705.0	training	3.75	1.00	-0.34
217	benzenemethanol, α-methyl-, acetate	93-92-5	62341	CC(C1=CC=CC=C1)OC(=O)C	1194	1312.0	test	6.75	6.08	-0.09
218	butanoic acid, 3-methyl-, butyl ester	109-19-3	7981	CCCCOC(=O)CC(C)C	1045	1080.8	training	5.75	2.70	-0.18
219	propanoic acid, 2-methyl-, 4-formyl-2-methoxyphenyl ester	20665-85-4	539829	CC(C)C(=O)OC1=C(C=C(C=C1)C=O)OC	1671	1579.0	training	9.50	2.94	-0.15
220	2(3H)-furanone, dihydro-5-propyl-	105-21-5	7742	CCCC1CCC(=O)O1	1154	1054.5	training	5.25	2.94	-0.13
221	butanoic acid	107-92-6	264	CCCC(=O)O	794	768.3	training	3.00	1.00	-0.09
222	1,2-cyclopentanedione, 3-methyl-	765-70-8	61209	CC1CCC(=O)C1=O	1027	1012.4	training	4.50	1.00	0.00
223	5H-5-methyl-6,7-dihydrocyclopentapyrazine	23747-48-0	32065	CC1CCC2=NC=CN=C12	1140	1237.7	training	6.50	2.50	-0.08
224	pyrazine, 2,3-diethyl-5-methyl-	18138-04-0	28905	CCC1=NC=C(N=C1CC)C	1155	1180.3	training	6.50	0.91	-0.13
225	furan, 2-methyl-5-(methylthio)-	13678-59-6	61657	CC1=CC=C(O1)SC	951	1020.8	training	4.50	1.00	0.01
226	pyrazine, 2-ethyl-3-methyl-	15707-23-0	27457	CCC1=NC=CN=C1C	1003	1034.6	training	5.50	1.31	-0.17
227	pyrazine, ethyl-	13925-00-3	26331	CCC1=NC=CN=C1	915	970.8	training	5.00	2.62	-0.20
228	thiazole, 5-ethenyl-4-methyl-	1759-28-0	15654	CC1=C(SC=N1)C=C	1026	1024.8	training	4.50	0.33	0.03
229	pyrazine, 2-methoxy-3-(2-methylpropyl)-	24683-00-9	32594	CC(C)CC1=NC=CN=C1OC	1181	1250.6	training	7.50	1.31	-0.23
230	4-methylthiazole	693-95-8	12748	CC1=CSC=N1	817	903.8	training	3.50	0.50	0.04
231	5-thiazoleethanol, 4-methyl-	137-00-8	1136	CC1=C(SC=N1)CCO	1277	1176.6	training	5.25	1.31	0.10
232	β-pinene	127-91-3	14896	CC1(C2CCC(=C)C1C2)C	978	1185.3	training	5.50	1.65	0.06
233	furan, 2-[(methylthio)methyl]-	1438-91-1	518937	CSCC1=CC=CO1	1001	1052.7	test	4.50	3.30	0.01
234	2-furfurylthiol	98-02-2	7363	C1=COC(=C1)CS	911	864.8	training	4.00	3.30	-0.19
235	pyrazine, tetramethyl-	1124-11-4	14296	CC1=C(N=C(C(=N1)C)C)C	1087	1093.7	training	6.00	0.00	-0.15
236	pyrazine, trimethyl-	14667-55-1	26808	CC1=CN=C(C(=N1)C)C	1004	1016.5	training	5.50	0.00	-0.17

237	pyrazine, 2-methyl-3-(methylthio)-	2882-20-4	76152	CC1=NC=CN=C1SC	1169	1130.7	training	5.50	1.00	-0.01
238	5,6,7,8-tetrahydroquinoxaline	34413-35-9	36822	C1CCC2=NC=CN=C2C1	1209	1287.8	training	6.50	6.12	-0.08
239	pyrazine, 2-methyl-6-(methylthio)-	2884-13-1	520398	CC1=CN=CC(=N1)SC	1187	1123.8	test	5.50	0.50	-0.01
240	ethanone, 1-(3-pyridinyl)-	350-03-8	9589	CC(=O)C1=CN=CC=C1	1111	1087.2	training	5.25	3.53	-0.09
241	2-acetyl-5-methylfuran	1193-79-9	14514	CC1=CC=C(O1)C(=O)C	1038	1027.1	training	5.25	1.00	-0.13
242	acetylpyrazine	22047-25-2	30914	CC(=O)C1=NC=CN=C1	1022	1049.5	test	5.75	1.65	-0.21
243	2-acetylthiazole	24295-03-2	520108	CC(=O)C1=NC=CS1	1019	1067.4	training	4.75	1.00	0.04
244	pyrazine, 2,3-dimethyl-	5910-89-4	22201	CC1=NC=CN=C1C	918	948.3	training	5.00	1.00	-0.20
245	creosol	93-51-6	7144	CC1=CC(=C(C=C1)O)OC	1193	1158.9	training	5.75	1.65	-0.03
246	2-ethyl-3-methoxypyrazine	25680-58-4	33135	CCC1=NC=CN=C1OC	1053	1081.3	training	6.50	1.31	-0.30
247	pyrazine, 2-ethyl-3,5-dimethyl-	13925-07-0	26334	CCC1=NC=C(N=C1C)C	1084	1098.3	test	6.00	0.33	-0.15
248	ethanone, 1-(2-pyridinyl)-	1122-62-9	14286	CC(=O)C1=CC=CC=N1	1033	1093.2	test	5.25	3.97	-0.09
249	benzene, 1,2-dimethoxy-4-(1-propenyl)-	93-16-3	637776	C/C=C/C1=CC(=C(C=C1)OC)OC	1457	1340.3	test	7.50	4.56	-0.16
250	phenol, 2-methyl-5-(1-methylethyl)-	499-75-2	10364	CC1=C(C=C(C=C1)C(C)C)O	1291	1217.6	training	5.75	1.65	0.06
251	hydrocoumarin	119-84-6	660	C1CC(=O)OC2=CC=CC=C21	1387	1350.7	training	6.75	6.63	-0.04
252	D-limonene	5989-27-5	440917	CC1=CC[C@@H](CC1)C(=C)C	1029	1107.6	training	5.00	3.30	0.00
253	eugenol	97-53-0	3314	COC1=C(C=CC(=C1)CC=C)O	1359	1332.5	test	6.75	4.56	-0.02
254	trans-isoeugenol	5932-68-3	853433	C/C=C/C1=CC(=C(C=C1)O)OC	1451	1332.5	training	6.75	4.56	-0.02
255	α-phellandrene	99-83-2	7460	CC1=CCC(C=C1)C(C)C	1006	1107.6	test	5.00	3.30	0.00
256	cyclohexane, 1-ethenyl-1-methyl-2,4-bis(1-methylethenyl)-,[1S-(1α,2β,4β)]-	515-13-9	6918391	CC(=C)[C@@H]1CC[C@@]([C@@H](C1)C(=C)C)(C)C=C	1426	1416.8	training	7.50	2.75	0.00
257	cyclohexene, 1-methyl-4-(1-methylethylidene)-	586-62-9	11463	CC1=CCC(=C(C)C)CC1	1089	1107.6	training	5.00	3.30	0.00
258	β-myrcene	123-35-3	31253	CC(=CCCC(=C)C=C)C	991	1002.0	test	4.50	3.15	-0.06
259	hexanoic acid, pentyl ester	540-07-8	10886	CCCCCC(=O)OCCCCC	1287	1310.7	test	6.75	8.61	-0.15
260	oxacyclohexadecan-2-one	106-02-5	235414	C1CCCCCCCOC(=O)CCCCCC1	1839	1920.3	training	9.25	25.78	-0.06
261	isophorone	78-59-1	6544	CC1=CC(=O)CC(C1)(C)C	1122	1114.4	training	5.25	1.50	0.00
262	eucalyptol	470-82-6	2758	CC1(C2CCC(O1)(CC2)C)C	1032	1283.8	training	6.50	3.30	-0.02

263	phenylethyl Alcohol	60-12-8	6054	C1=CC=C(C=C1)CCO	1114	1214.5	test	4.75	9.56	0.08
264	anisyl propionate	7549-33-9	61417	CCC(=O)OCC1=CC=C(C=C1)OC	1514	1447.8	training	8.25	5.49	-0.16
265	decanoic acid, ethyl ester	110-38-3	8048	CCCCCCCCCC(=O)OCC	1394	1427.1	test	7.25	11.86	-0.13

Table S3. Details of the curated database of metabolites identified in coffee and plantain by-product flours using gas chromatography–time-of-flight mass spectrometry (GC-MS-QTOF) in the HP-5ms capillary column, used to predict the retention index by means of the QSRR model. The Table includes the chemical name, CAS registry number, PubChem CID, SMILES, retention indices, applicability domain (AD) assessment and numerical values of the three conformation-independent molecular descriptors.

No.	Chemical name	CAS number	PubChem CID	SMILES	Retention indices			Inside the AD	Molecular descriptors		
					Experimental	Average	Predicted		Eta_betaS	MDEC-22	MATS1p
1	erythritol	149-32-6	222285	C([C@H]([C@H](CO)O)O)O	1193	1193	1072.5	yes	4.50	0.33	0.11
2	2,3-butanediol	513-85-9	262	CC(C(C)O)O	709	709	789.0	yes	3.00	0.00	-0.03
3	glycerol	56-81-5	753	C(C(CO)O)O	960	960	861.1	yes	3.25	0.50	0.03
4	arabitol	488-82-4	94154	C([C@H](C([C@@H](CO)O)O)O)O	1409	1409	1263.1	yes	5.75	0.25	0.16
5	diglycerol	627-82-7	42953	C(C(COCC(CO)O)O)O	1428	1428	1242.1	yes	6.50	1.98	-0.06
6	ethanolamine	141-43-5	700	C(CO)N	952	952	640.0	yes	2.00	1.00	-0.08
7	glycine	56-40-6	750	C(C(=O)O)N	993	993	777.4	yes	2.75	0.00	0.01
8	cycloleucine	52-52-8	2901	C1CCC(C1)(C(=O)O)N	1046	1046	1258.0	yes	5.25	4.24	0.17
9	glutamic acid	56-86-0	33032	C(CC(=O)O)[C@@H](C(=O)O)N	1210	1210	1257.2	yes	5.75	1.00	0.14
10	glucosamine-phosphate	2152-75-2	188960	C([C@@H]1[C@H]([C@@H]([C@H]([C@H](O1)OP(=O)(O)O)N)O)O)O	1492	1492	1685.8	no	10.75	0.00	-0.19
11	galactosamine	90-76-6	24154	C([C@@H]1[C@@H]([C@@H]([C@H](C(O1)O)N)O)O)O	1551	1551	1516.4	no	7.75	0.00	0.16
12	tyramine	51-67-2	5610	C1=CC(=CC=C1CCN)O	1604	1604	1325.1	yes	5.50	6.68	0.18
13	glucosaminic acid	3646-68-2	73563	C([C@H]([C@H]([C@@H]([C@H](C(=O)O)N)O)O)O)O	1685	1685	1567.6	no	7.75	0.00	0.24
14	valine	72-18-4	6287	CC(C)[C@@H](C(=O)O)N	764	764	989.8	yes	4.25	0.00	0.04
15	valeramide	626-97-1	12298	CCCCC(=O)N	823	823	880.0	yes	3.50	2.38	-0.03
16	isoleucine	443-79-8	791	CCC(C)C(C(=O)O)N	858	858	1052.8	yes	4.75	0.00	0.04
17	serine	56-45-1	5951	C([C@@H](C(=O)O)N)O	941	941	1013.9	yes	4.00	0.00	0.13
18	pipecolic acid	535-75-1	849	C1CCNC(C1)C(=O)O	948	948	1192.0	yes	5.50	3.97	0.02
19	threonine	72-19-5	6288	C[C@H]([C@@H](C(=O)O)N)O	979	979	1070.3	yes	4.50	0.00	0.12

20	malonamide	108-13-4	7911	C(C(=O)N)C(=O)N	1078	1078	1022.4	yes	4.00	0.00	0.14
21	acetyl-glutamic acid	1188-37-0	70914	CC(=O)N[C@@H](CCC(=O)O)C(=O)O	1179	1179	1420.4	yes	7.75	1.00	-0.01
22	ornithine	70-26-8	6262	C(C[C@@H](C(=O)O)N)CN	1271	1271	1173.0	yes	5.00	2.38	0.13
23	2-Deoxy-ribose	533-67-5	5460005	C(C=O)[C@@H]([C@@H](CO)O)O	1226	1226	1127.8	yes	5.00	1.31	0.08
24	deoxyglucose	154-17-6	108223	C(C=O)[C@H]([C@@H]([C@@H](CO)O)O)O	1448	1448	1315.6	yes	6.25	1.11	0.13
25	1,5-anhydroglucitol	154-58-5	64960	C1[C@@H]([C@H]([C@@H]([C@H](O1)CO)O)O)O	1187	1349	1380.9	yes	7.00	0.33	0.09
	1,5-anhydrosorbitol				1511						
26	methyl-galactopyranoside	3396-99-4	76935	CO[C@@H]1[C@@H]([C@H]([C@H]([C@H](O1)CO)O)O)O	1528	1528	1480.4	yes	8.50	0.00	-0.05
27	galactonic acid	576-36-3	128869	C([C@H]([C@@H]([C@@H]([C@H](C(=O)O)O)O)O)O)O	1549	1549	1556.4	no	7.75	0.00	0.23
28	sorbose	87-79-6	439192	C1[C@@H]([C@H]([C@@H](C(O1)(CO)O)O)O)O	1546	1564	1508.7	no	7.75	0.33	0.14
	fructose	57-48-7	2723872	C1[C@H]([C@H]([C@@H](C(O1)(CO)O)O)O)O	1582						
29	galactose	59-23-4	6036	C([C@@H]1[C@@H]([C@@H]([C@H](C(O1)O)O)O)O)O	1551	1570	1504.4	no	7.75	0.00	0.14
	mannose	3458-28-4	18950	C([C@@H]1[C@H]([C@@H]([C@@H](C(O1)O)O)O)O)O	1551						
	glucose	2280-44-6	5793	C([C@@H]1[C@H]([C@@H]([C@H](C(O1)O)O)O)O)O	1608						
30	sedoheptulose anhydride monohydrate	469-90-9	16219957	C1[C@@H]2[C@H]([C@H]([C@@H]([C@](O1)(O2)CO)O)O)O)O	1599	1599	1625.1	no	9.00	0.33	0.07
31	glucoheptonic acid	87-74-1	25588	C([C@H]([C@H]([C@@H]([C@H]([C@H](C(=O)O)O)O)O)O)O)O	1712	1712	1728.6	no	9.00	0.00	0.24
32	mucic acid	526-99-8	3037582	[C@@H]([C@@H]([C@H](C(=O)O)O)O)([C@@H](C(=O)O)O)O	1717	1717	1674.9	no	8.50	0.00	0.26
33	xylose	25990-60-7	135191	C1[C@H]([C@@H]([C@H](C(O1)O)O)O)O	1313	1332.5	1317.8	yes	6.50	0.00	0.10
	ribose	50-69-1	10975657	C1[C@H]([C@H]([C@H](C(O1)O)O)O)O	1352						
34	palmitic acid	57-10-3	985	CCCCCCCCCCCCCCCC(=O)O	1722	1722	1867.6	yes	9.00	23.27	-0.02
35	nonanoic acid	112-05-0	8158	CCCCCCCCC(=O)O	1039	1039	1228.3	yes	5.50	9.33	-0.03

36	heptadecanoic acid	506-12-7	10465	CCCCCCCCCCCCCCCCC(=O)O	1819	1819	1959.2	no	9.50	25.33	-0.02
37	2-furoic acid	88-14-2	6919	C1=COC(=C1)C(=O)O	813	813	1071.4	yes	5.00	2.38	-0.04
38	malonic acid	141-82-2	867	C(C(=O)O)C(=O)O	887	887	965.3	yes	4.00	0.00	0.05
39	fumaric acid	110-17-8	444972	C(=C/C(=O)O)\C(=O)O	1025	1025	1086.5	yes	4.50	1.00	0.12
40	hydroxyhexanoic acid	1191-25-9	14490	C(CCC(=O)O)CCO	1083	1083	1111.5	yes	4.75	5.68	0.01
41	malic acid	6915-15-7	525	C(C(C(=O)O)O)C(=O)O	1177	1177	1177.0	yes	5.25	0.00	0.13
42	hydroxyglutaric acid	2889-31-8	43	C(CC(=O)O)C(C(=O)O)O	1263	1263	1240.5	yes	5.75	1.00	0.11
43	aconitic acid	585-84-2	643757	C(/C(=C/C(=O)O)/C(=O)O)C(=O)O	1434	1434	1435.1	no	7.00	0.50	0.18
44	dihydroxybenzoic acid	99-50-3	72	C1=CC(=C(C=C1C(=O)O)O)O	1511	1511	1468.5	no	6.50	1.65	0.31
45	quinic acid	77-95-2	6508	C1[C@H](C([C@@H](CC1(C(=O)O)O)O)O)O	1568	1568	1634.2	no	8.00	0.50	0.29
46	gluconic acid lactone	90-80-2	7027	C([C@@H]1[C@H]([C@@H]([C@H](C(=O)O1)O)O)O)O	1581	1581	1487.5	yes	7.75	0.00	0.11
47	glucuronolactone	32449-92-6	92283	C(=O)[C@@H]([C@@H]1[C@@H]([C@@H](C(=O)O1)O)O)O	1608	1608	1465.1	yes	7.75	0.00	0.08
48	gallic acid	149-91-7	370	C1=C(C=C(C(=C1O)O)O)C(=O)O	1653	1653	1597.4	no	7.25	0.50	0.38
49	shikimic acid	138-59-0	8742	C1[C@H]([C@@H]([C@@H](C=C1C(=O)O)O)O)O	1905	1905	1528.3	no	7.25	0.50	0.27
50	pyruvic acid	127-17-3	1060	CC(=O)C(=O)O	725	725	785.4	yes	3.25	0.00	-0.08
51	lactic acid	79-33-4	107689	C[C@@H](C(=O)O)O	738	738	829.6	yes	3.25	0.00	-0.01
52	glycolic acid	79-14-1	757	C(C(=O)O)O	753	753	732.8	yes	2.75	0.00	-0.07
53	oxalic acid	144-62-7	971	C(=O)(C(=O)O)O	812	812	907.8	yes	3.50	0.00	0.06
54	hydroxybutyric acid	300-85-6	441	CC(CC(=O)O)O	842	842	904.1	yes	3.75	0.00	0.00
55	nicotinic acid	59-67-6	938	C1=CC(=CN=C1)C(=O)O	975	975	1192.8	yes	5.50	3.53	0.03
56	succinic acid	110-15-6	1110	C(CC(=O)O)C(=O)O	994	994	1037.1	yes	4.50	1.00	0.04
57	glyceric acid	473-81-4	752	C(C(C(=O)O)O)O	1017	1017	988.1	yes	4.00	0.00	0.09
58	dihydroxymalonic acid	560-27-0	68412	C(=O)(C(C(=O)O)(O)O)O	1121	1121	1292.3	no	5.50	0.00	0.27
59	3-hydroxypropanoic acid	503-66-2	68152	C(CO)C(=O)O	1250	1250	842.5	yes	3.25	1.00	-0.01
60	hydroxybenzoic acid	99-96-7	135	C1=CC(=CC=C1C(=O)O)O	1313	1313	1339.4	no	5.75	3.30	0.22
61	vanillic acid	121-34-6	8468	COC1=C(C=CC(=C1)C(=O)O)O	1452	1452	1406.5	yes	7.25	1.65	0.05

62	gulonic acid lactone	1128-23-0	439373	C([C@@H]([C@@H]1[C@@H]([C@@H](C(=O)O1)O)O)O)O	1601	1601	1487.5	yes	7.75	0.00	0.11
63	caffeine	58-08-2	2519	CN1C=NC2=C1C(=O)N(C(=O)N2C)C	1540	1540	1524.1	no	10.75	0.00	-0.45
64	1,2-dihydro-1,2-naphthalenediol	31966-70-8	119261	C1=CC=C2[C@@H]([C@@H](C=CC2=C1)O)O	1313	1313	1530.4	yes	7.00	6.63	0.20
65	4-O-methylphloracetophenone	7507-89-3	24135	CC(=O)C1=C(C=C(C=C1O)OC)O	1398	1398	1447.6	yes	7.75	0.50	0.04
66	1,3-dihydroxyacetone	96-26-4	670	C(C(=O)CO)O	926	926	836.0	yes	3.25	0.50	-0.01
67	phosphoric acid	7664-38-2	1004	OP(=O)(O)O	960	960	545.3	no	3.00	0.00	-0.42

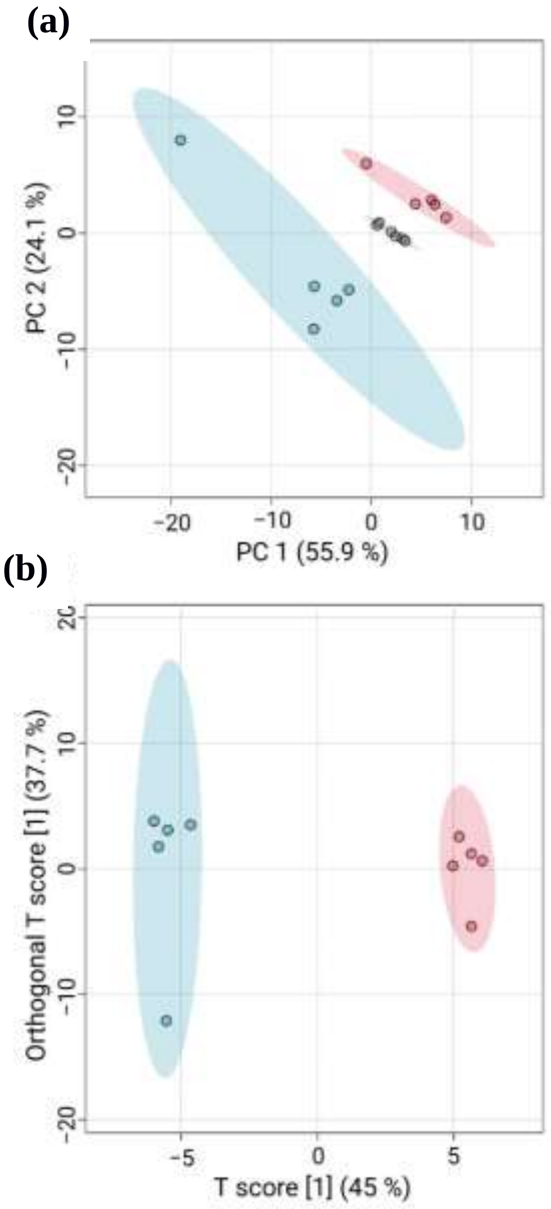

Figure S1. Multivariate analysis of control and extruded coffee and plantain by-product flour samples. (a) Principal Component Analysis (explained variance of 80 %); (b) Orthogonal Partial Least Squares Discriminant Analysis, R^2: 0.88 and Q^2: 0.84. Samples from control are shown in blue, extruded flour in red, and quality control (QC) samples in gray.
